# Supplementary material for: Identifying sorafenib benefit among patients with hepatocellular carcinoma: A transcriptomic and genomic approach
Source: JHEP Rep. 2026 Jan 27;8(4):101742. doi: 10.1016/j.jhepr.2026.101742 (PMC12969672; doi:10.1016/j.jhepr.2026.101742)
Supplement: Multimedia component 1 [file mmc1.pdf]

Identifying sorafenib benefit among patients with hepatocellular carcinoma: A transcriptomic and genomic approach

Sun Young Yim, Hayeon Kim, Tae Hyung Kim, Sang-Hee Kang, Youngwoo Lee, Eunho Choi, Yang Jae Yoo, Seong Hee Kang, Young-Sun Lee, Young Kul Jung, Yeon Seok Seo, Hyung Joon Yim, Jong Eun Yeon, Kyung Suk Yang, Yitao Tang, Bowha Sohn, Yun Seong Jeong, Hyewon Park, Han Liang, Ju-Seog Lee, Ji Hoon Kim

Table of contents

Fig. S1……………………………………………………………………..………………………2
Fig. S2……………………………………………………………………..………………………3
Fig. S3……………………………………………………………………..………………………4
Fig. S4……………………………………………………………………..………………………5
Fig. S5……………………………………………………………………..………………………6
Fig. S6……………………………………………………………………..………………………7
Fig. S7……………………………………………………………………..………………………8
Fig. S8……………………………………………………………………..………………………9
Fig. S9……………………………………………………………………..……………………10
Table S1……………………………………………………………………………………………11
Table S2……………………………………………………………………………………………13
Table S3……………………………………………………………………………………………15
Table S4……………………………………………………………………………………………16
Table S5……………………………………………………………………………………………17
Table S6……………………………………………………………………………………………18

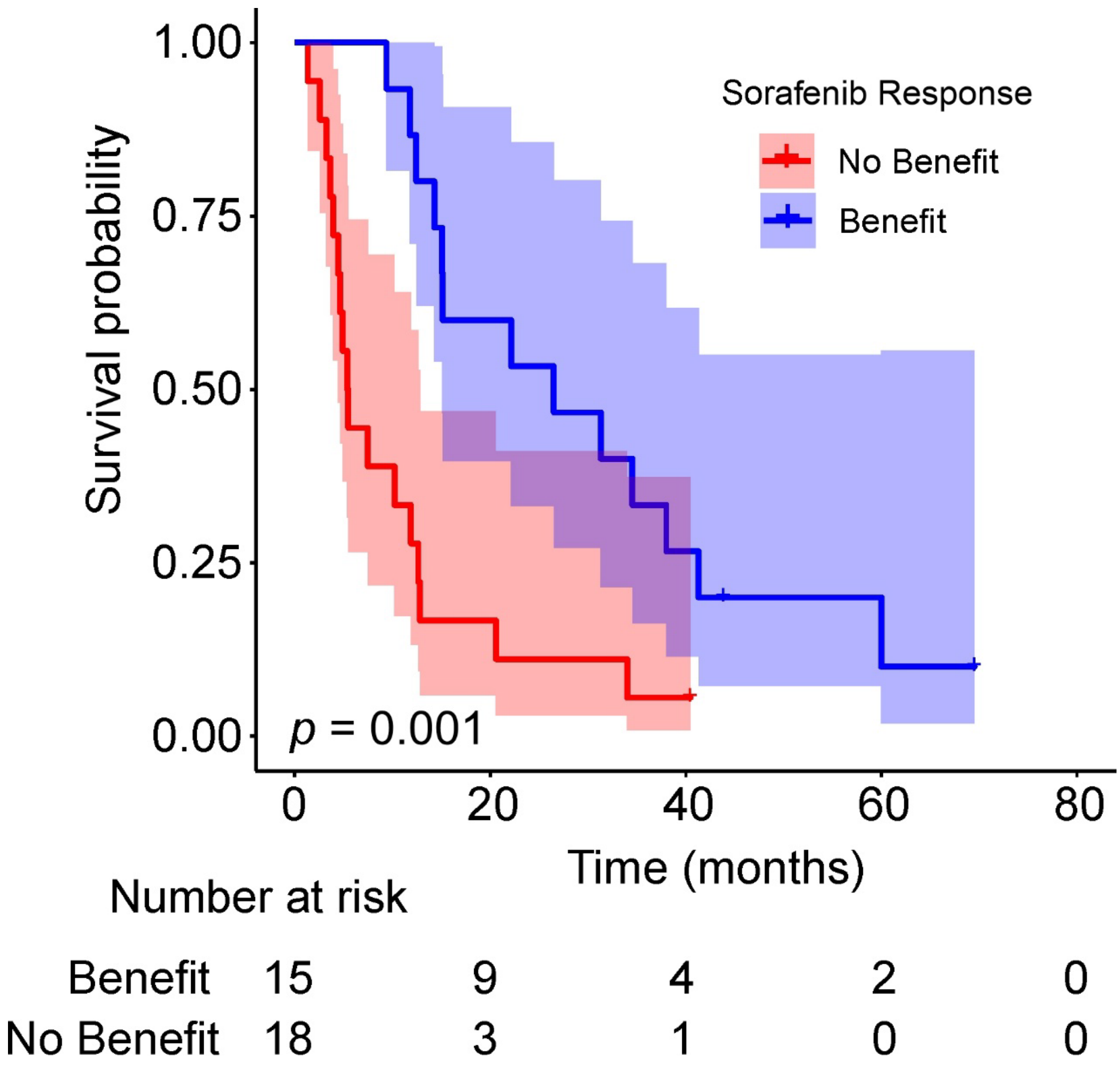

Fig. S1. Overall survival differs significantly between the sorafenib benefit and non-benefit groups in the KU cohort.

Kaplan–Meier curves comparing overall survival (OS) between patients in the KU cohort classified as having a sorafenib benefit (n = 15) or no sorafenib benefit (n = 18) based on clinical response to sorafenib treatment. The benefit group included patients who did not experience disease progression while receiving sorafenib, whereas the non-benefit group comprised patients whose disease progressed despite treatment. A significant difference in OS was observed between the two groups (log-rank p = 0.001), with the benefit group showing markedly improved survival. These findings highlight the clinical importance of distinguishing patients who derive benefit from sorafenib and provide a basis for developing predictive gene expression signatures.

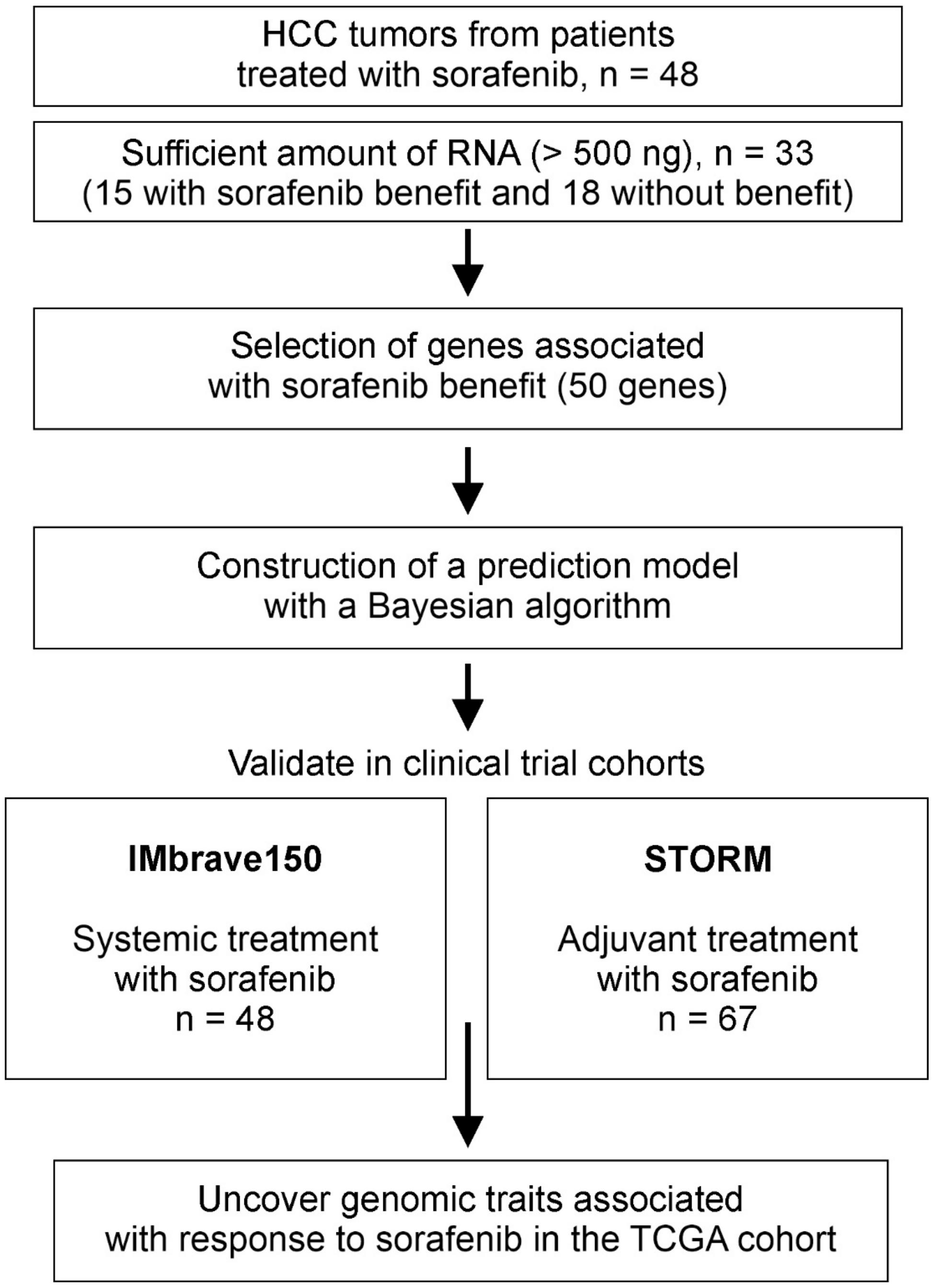

Fig. S2. Schematic of the data analysis for the identification and confirmation of predictive biomarkers in sorafenib-treated HCC patients.

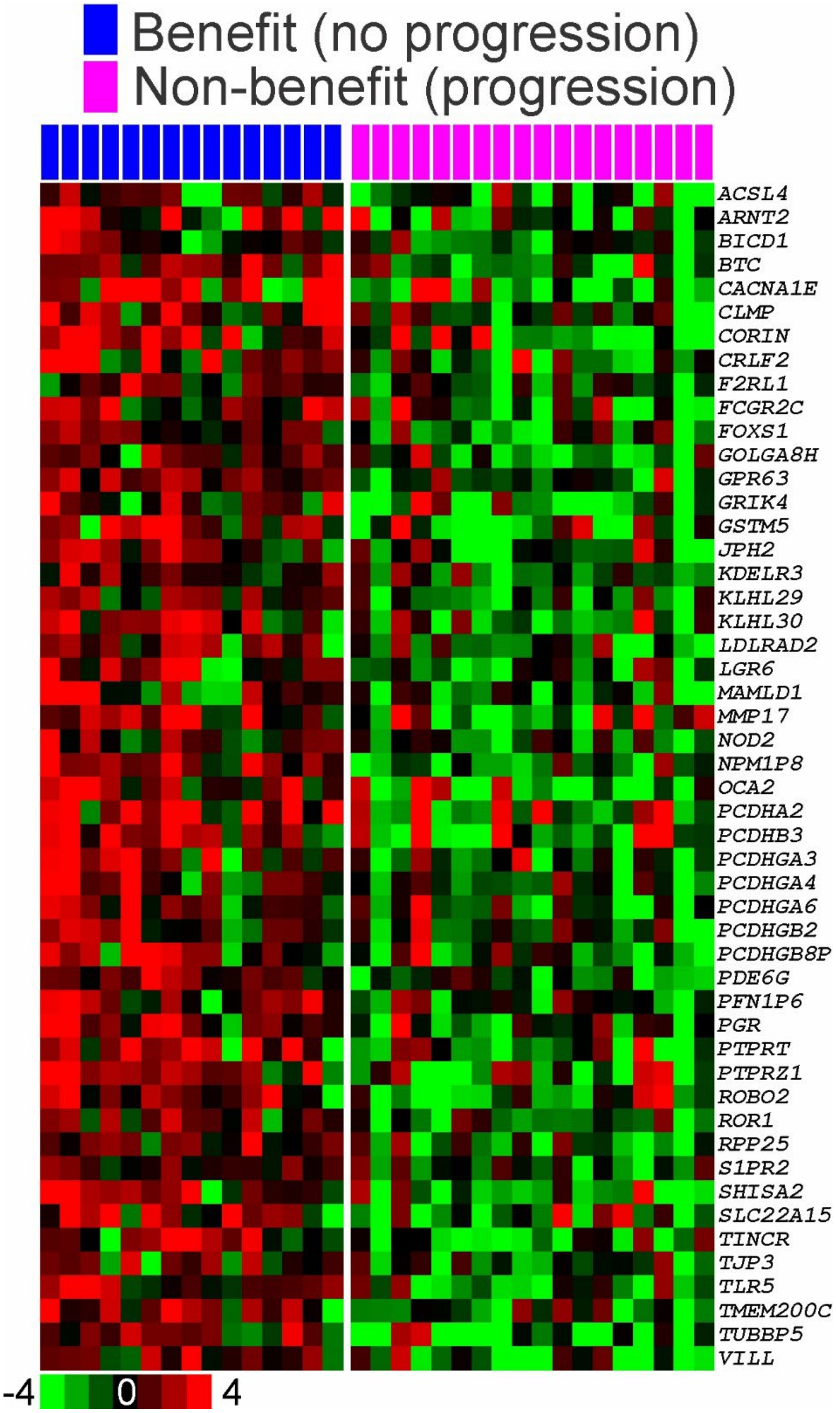

Fig. S3. Expression patterns of the genes included in the KUSS50.

The data are presented in matrix format; each row represents an individual gene, and each column represents a different tissue sample. Each cell in the matrix represents the expression level of a gene in an individual tissue sample. The red and green colors in the cells reflect relatively high and low gene expression levels, respectively, as indicated by the scale bar (log2-transformed scale).

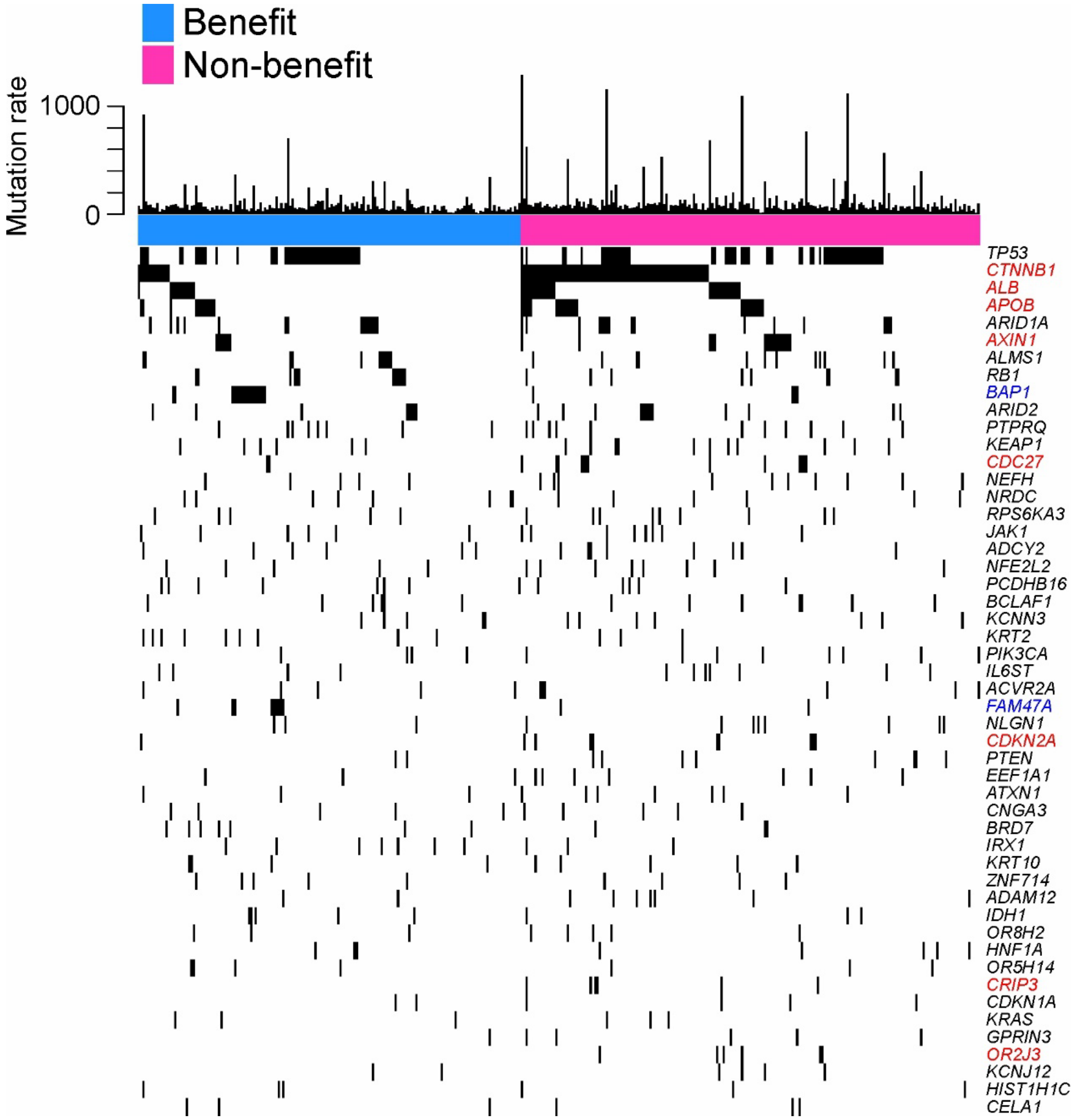

Fig. S4. Profile of the somatic mutations in the KUSS50-defined subtypes in the TCGA cohort. The top panel displays the individual tumor mutation rates for each patient in the TCGA cohort. Each horizontal bar represents a single tumor sample, with the height of the bar indicating the overall mutation rate (or tumor mutation burden) for that tumor. The bottom panel shows the mutation landscape, with genes exhibiting significant levels of mutation in the TCGA cohort highlighted. The genes displayed in this panel met two criteria: 1) a false discovery rate (FDR) of less than 0.1, as determined via MutSig suite analysis, and 2) a mutation rate greater than 7% across the cohort. The gene symbols highlighted in red indicate genes that are significantly associated with the non-benefit subtype, as determined by a chi-square test ($p < 0.05$). Conversely, the gene symbols highlighted in blue represent genes significantly associated with the benefit subtype.

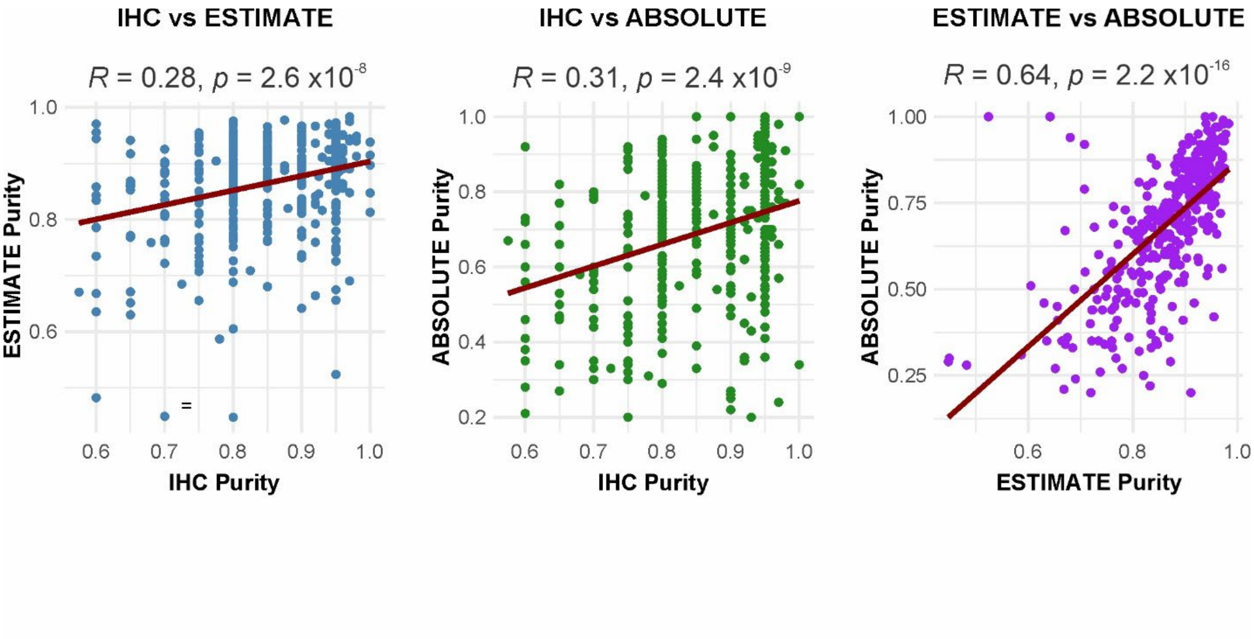

Fig. S5. Correlation among the three independent methods used to estimate the purity of HCC tumors from the TCGA cohort.

The strength and significance of the correlations were assessed using the Pearson correlation test, with corresponding *R* and *p*-values indicated on each plot.

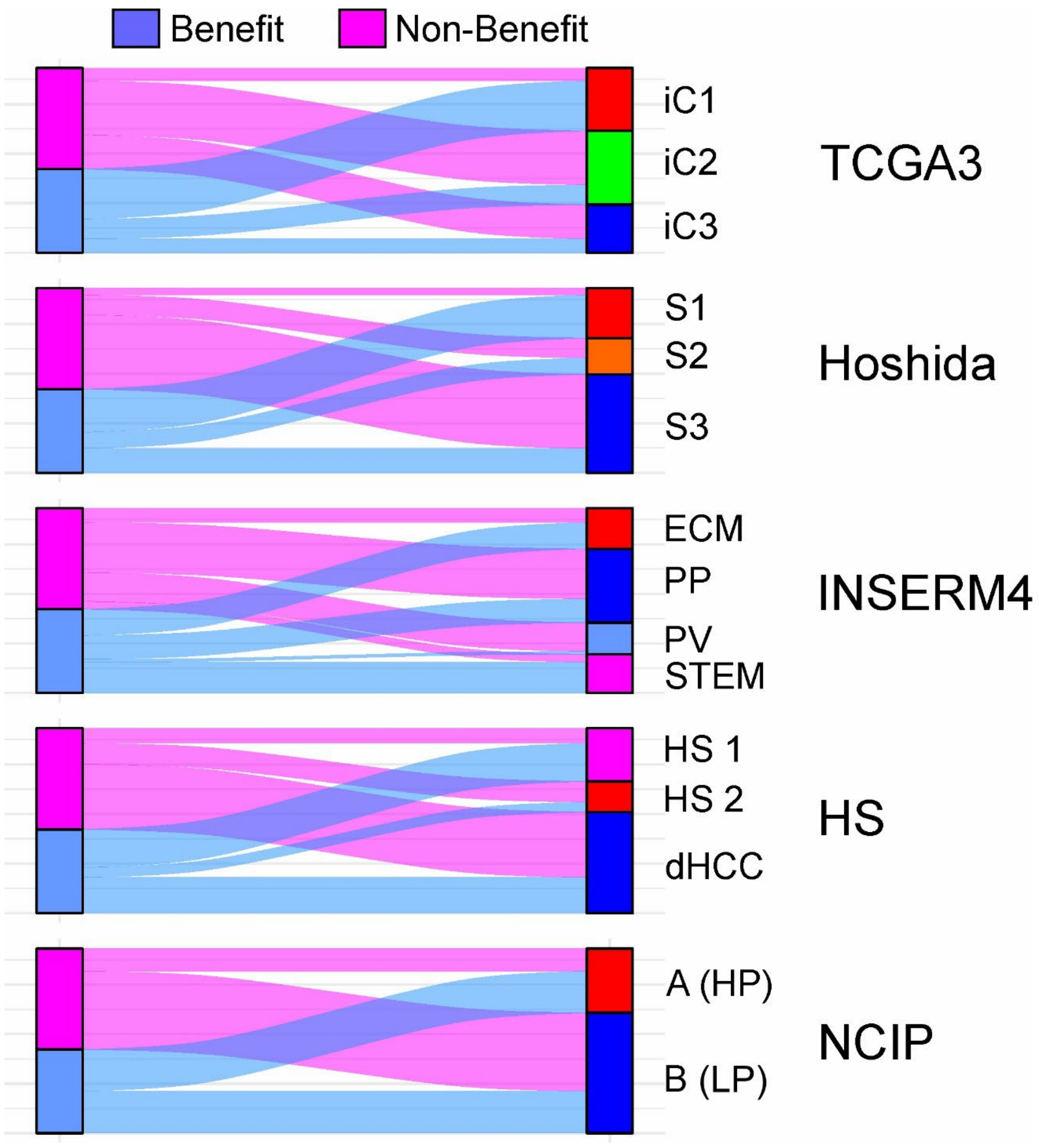

Fig. S6. Sankey plots of the KUSS50-defined subtypes compared with genomic subtypes previously identified in HCC patients.

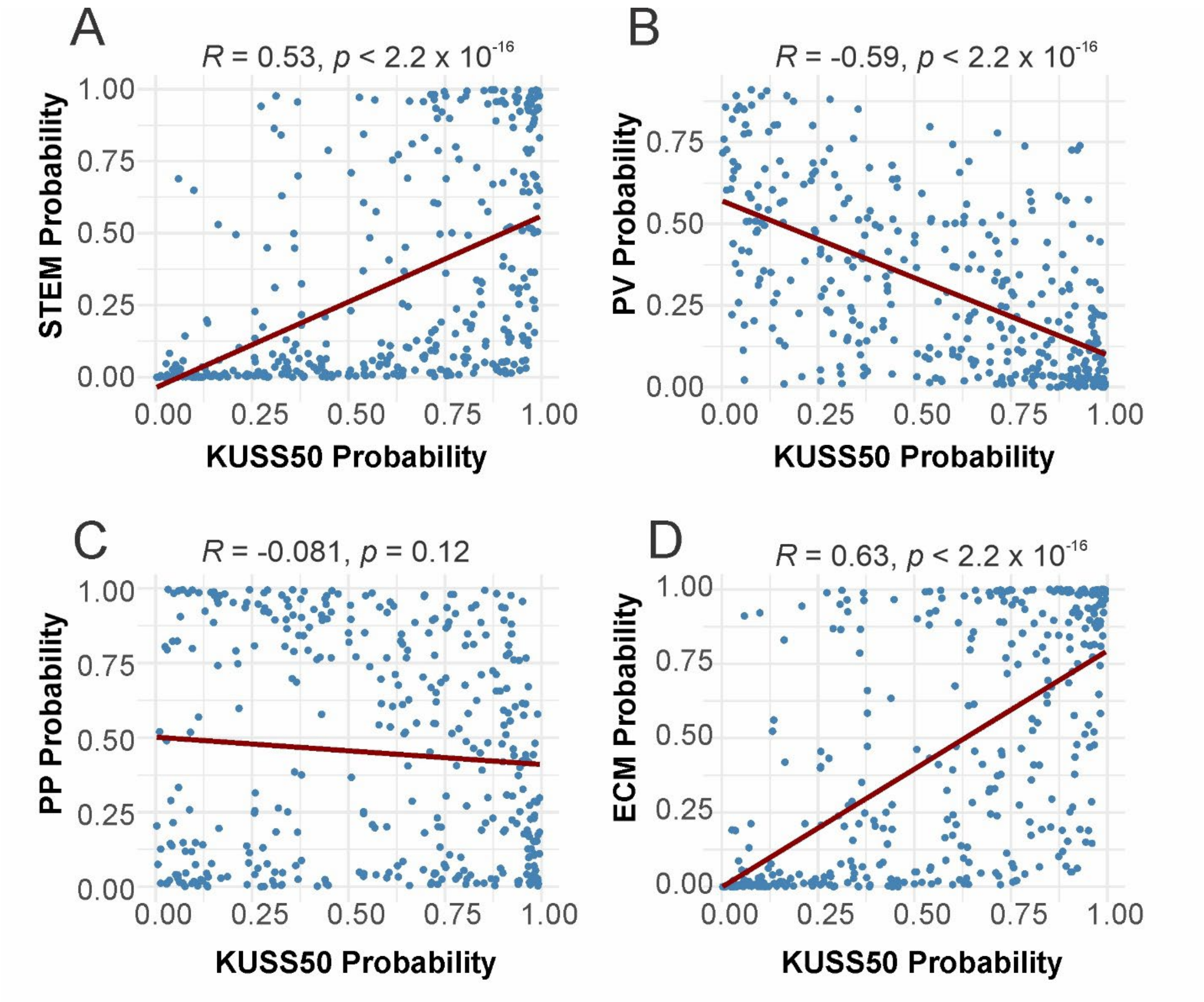

Fig. S7. Correlation of KUSS50-based subtype probability with the probabilities of four INSERM molecular subtypes in HCC.

(A) STEM (stem cell), (B) PV (perivenous), (C) PP (periportal), and (D) ECM (extracellular matrix) subtype probabilities were plotted against KUSS50 probability scores. Each dot represents an individual HCC tumor. The red line indicates the linear regression (lm) fit, and Pearson correlation coefficients (*R*) with associated *p*-values are shown. Significant positive correlations were observed between the KUSS50-defined subtype and the STEM subtype (*R* = 0.53) and ECM subtype (*R* = 0.63), while a significant negative correlation was seen with the PV subtype (*R* = -0.59). No significant correlation was found with the PP subtype. Statistical significance was estimated by the Pearson correlation test.

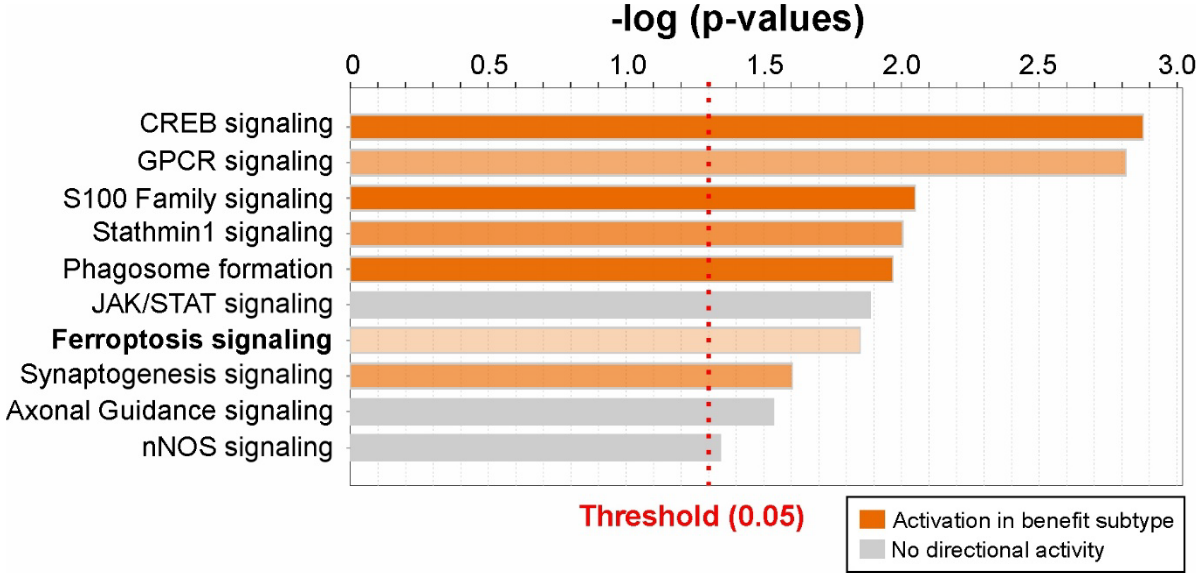

Fig. S8. Ingenuity Pathway Analysis (IPA) identifies the canonical pathways of genes in the KUSS50.

The x-axis represents the –log(p) value of pathway enrichment, and the y-axis lists the 10 most significantly enriched canonical pathways among genes upregulated in the KUSS50-defined benefit subtype. Each bar corresponds to a canonical pathway, with its length indicating the level of statistical significance of enrichment. Pathway enrichment significance was determined using the right-tailed Fisher exact test; longer bars indicate stronger enrichment, i.e., higher –log(p) values. The color of each bar denotes the activation *z*-score, reflecting predicted activation (orange), inhibition (blue), or undetermined directionality (gray) based on expression patterns. Pathways with $p < 0.05$ were considered significantly enriched.

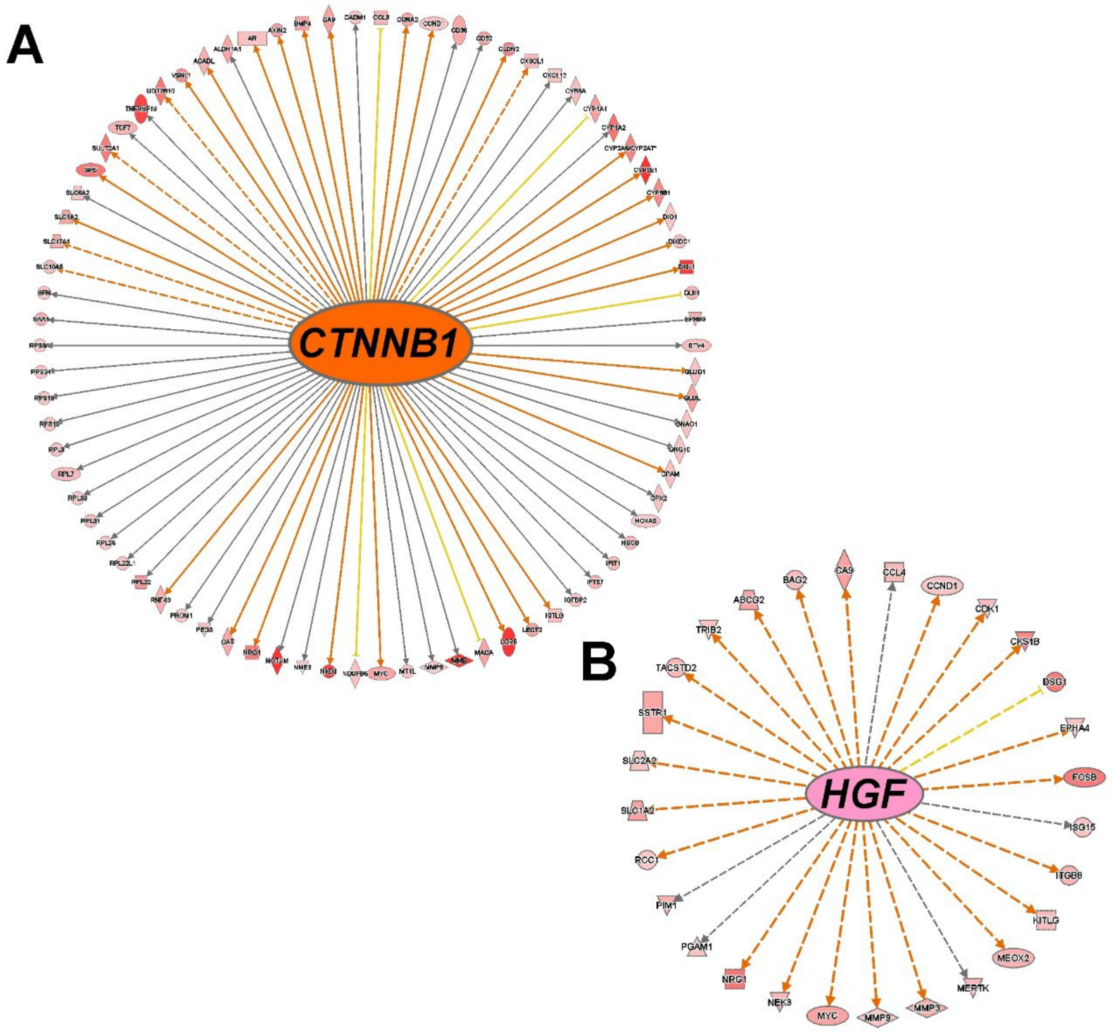

Fig. S9. Gene networks of upstream regulators activated in the non-benefit subtype of HCC.

Upstream regulator analysis was performed using Ingenuity Pathway Analysis (IPA) of the genes upregulated in the non-benefit subtype of HCC. Shown here are the gene networks centered on key upstream regulators predicted to be significantly activated based on the *z*-scores and expression patterns of downstream targets. (A) The CTNNB1 (β-catenin) network showing a broad range of transcriptional targets, reflecting its central role in Wnt signaling and previously known association with sorafenib resistance. (B) The HGF (hepatocyte growth factor) network illustrating the activation of mitogenic and pro-survival signaling pathways. Node colors represent expression changes (e.g., red for upregulated targets), and edge colors indicate the direction and type of regulation inferred by IPA. Solid and dashed lines represent direct and indirect relationships, respectively.

Table S1. Clinical characteristics of HCC patients treated with sorafenib.

Clinical characteristic	Total	Benefit	Non-benefit	*p*-value
**No. of patients**	33	15	18	
**Age, years, median (range)**	58 (32-83)	58 (41-71)	57 (32-83)	0.915
**Gender, male, *N* (%)**	27 (81.8)	12 (80)	15 (83.3)	0.805
**Cirrhosis, *N* (%)**	19 (57.6)	8 (53.3)	11 (61.1)	0.653
**AST, IU/L, median (range)**	55 (19-317)	41 (19-157)	38 (19-317)	0.957
**ALT, IU/L, median (range)**	29 (7-211)	29 (14-90)	29 (7-211)	0.492
**Total bilirubin, mg/dL, median (range)**	0.6 (0.33-5.07)	0.59 (0.36-2.02)	0.7 (0.33-5.07)	0.575
**Albumin, median (range)**	2.9 (2.9-4.6)	4.2 (3.1-4.4)	4.0 (2.9-4.6)	0.284
**INR, median (range)**	1.03 (0.89-1.23)	1.02 (0.89-1.19)	1.05 (0.92-1.23)	0.302
**CTP grade**				0.538
**5**	25	14 (77.8)	11 (73.3)	
**6**	7	3 (16.7)	4 (26.7)	
**8**	1	1 (5.6)	0	
**ALBI score, median (range)**	-2.74 (-3.23- -1.44)	-2.80 (-3.21- -1.82)	-2.67 (-3.23- -1.44)	0.29
**Log_{10} AFP, median (range)**	2.17 (0.04-6.28)	1.76 (0.34-6.28)	2.46 (0.04-5.68)	0.708
**Log_{10}PIVKA, median (range)**	2.33 (0.95-5)	1.58 (0.95-5.00)	2.46 (1.08-5.00)	0.156
**Etiology, *N* (%)**				
HBV	24 (72.7)	12 (80)	12 (66.7)	0.539
HCV	1 (3)	0	1 (5.6)	
Nonviral	8 (24.2)	3 (20)	5 (27.8)	
**Extrahepatic metastasis, *N* (%)**	29 (87.9)	12 (80)	17 (94.4)	0.206
**Vessel invasion, *N* (%)**				
Major portal vein	8 (24.2)	3 (20)	5 (27.8)	0.604
Major hepatic vein	3 (9.1)	2 (13.3)	1 (5.6)	0.439
**Performance status, *N* (%)**				0.730

0	23 (69.7)	10 (66.7)	13 (72.2)	
1	10 (30.3)	5 (33.3)	5 (27.8)	
**BCLC stage, *N* (%)**				0.894
B	2 (6.1)	1 (6.7)	1 (5.6)	
C	31 (93.9)	14 (93.3)	17 (94.4)	
**Dermatologic adverse event, *N* (%)**	17 (51.5)	11(73.3)	6 (33.3)	0.022
**2nd-line CTx, *N* (%)**				
None	14 (42.4)	7 (46.7)	7 (38.9)	0.566
APX	8 (24.2)	4 (26.7)	4 (22.2)	
EPUL	4 (12.1)	1 (6.7)	3 (16.7)	
Nivolumab	2 (6.1)	0	2 (11.1)	
Regorafenib	5 (15.2)	3 (20)	2 (11.1)	

AST, aspartate aminotransferase; ALT, alanine aminotransferase; INR, international normalized ratio; CTP, Child-Pugh Score; ALBI, albumin-bilirubin; AFP, alpha-fetoprotein; PIVKA, proteins induced by vitamin K absence or antagonism-II; HBV, hepatitis B virus; HCV, hepatitis C virus; BCLC, Barcelona Clinic Liver Cancer; CTx, chemotherapy; APX, adriamycin + capecitabine; EPUL, epirubicin, cisplatin, UFT, and leucovorin

Table S2. Genes in the KUSS50.

EnsEMBL gene ID	Symbol	Ratio (benefit/non-benefit)
ENSG00000068366	*ACSL4*	1.69
ENSG00000172379	*ARNT2*	2.09
ENSG00000151746	*BICD1*	1.04
ENSG00000174808	*BTC*	2.10
ENSG00000198216	*CACNA1E*	2.58
ENSG00000166250	*CLMP*	2.01
ENSG00000145244	*CORIN*	2.37
ENSG00000205755	*CRLF2*	1.72
ENSG00000164251	*F2RL1*	1.20
ENSG00000244682	*FCGR2C*	1.55
ENSG00000179772	*FOXS1*	1.51
ENSG00000261794	*GOLGA8H*	1.72
ENSG00000112218	*GPR63*	1.74
ENSG00000149403	*GRIK4*	2.28
ENSG00000134201	*GSTM5*	2.27
ENSG00000149596	*JPH2*	1.61
ENSG00000100196	*KDELR3*	1.15
ENSG00000119771	*KLHL29*	1.74
ENSG00000168427	*KLHL30*	1.78
ENSG00000187942	*LDLRAD2*	1.67
ENSG00000133067	*LGR6*	1.65
ENSG00000013619	*MAMLD1*	1.45
ENSG00000198598	*MMP17*	1.71
ENSG00000167207	*NOD2*	1.21
ENSG00000236285	*NPM1P8*	2.54
ENSG00000104044	*OCA2*	2.96
ENSG00000204969	*PCDHA2*	1.93
ENSG00000113205	*PCDHB3*	2.00
ENSG00000254245	*PCDHGA3*	1.98
ENSG00000262576	*PCDHGA4*	1.46
ENSG00000253731	*PCDHGA6*	1.42
ENSG00000253910	*PCDHGB2*	1.17
ENSG00000248449	*PCDHGB8P*	1.55
ENSG00000185527	*PDE6G*	1.95
ENSG00000227212	*PFN1P6*	1.45
ENSG00000082175	*PGR*	2.04
ENSG00000196090	*PTPRT*	2.21
ENSG00000106278	*PTPRZ1*	2.37
ENSG00000185008	*ROBO2*	1.76
ENSG00000185483	*ROR1*	1.56

ENSG00000178718	*RPP25*	1.70
ENSG00000267534	*S1PR2*	1.04
ENSG00000180730	*SHISA2*	2.35
ENSG00000163393	*SLC22A15*	1.83
ENSG00000223573	*TINCR*	2.01
ENSG00000105289	*TJP3*	1.13
ENSG00000187554	*TLR5*	2.11
ENSG00000206432	*TMEM200C*	1.65
ENSG00000159247	*TUBBP5*	2.85
ENSG00000136059	*VILL*	1.85

Table S3. Factors associated with benefit from sorafenib treatment.

Characteristic	Univariate		Multivariate	
	HR (95% CI)	*p*-value	HR (95% CI)	*p*-value
Age, years	1.011 (0.971-1.052)	0.606		
Male gender	0.507 (0.199-1.291)	0.154		
Liver cirrhosis	0.917 (0.446-1.882)	0.813		
CTP grade	1 (0.422-2.366)	1		
ALBI score	2.412 (0.957-6.078)	0.062		
Log_{10} AFP	1.206 (0.964-1.509)	0.101	1.348 (1.048-1.733)	0.02
Log_{10}PIVKA	1.302 (0.95-1.783)	0.1		
Etiology				
Non-HBV	Reference			
HBV	1.408 (0.639-3.102)	0.396		
Extrahepatic metastasis				
No	Reference			
Yes	2.709 (0.628-11.685)	0.181		
Major portal vein	0.874 (0.371-2.058)	0.757		
Major hepatic vein	1.025 (0.316-3.517)	0.935		
ECOG performance status				
0	Reference		Reference	
1	1.935 (0.884-4.238)	0.099	2.862 (1.193-6.863)	0.018
BCLC stage				
B	Reference			
C	1.416 (0.316-6.351)	0.649		
Skin adverse event	0.422 (0.199-0.894)	0.024		
KUSS50				
Benefit	Reference		Reference	
Non-benefit	0.288 (0.132-0.633)	0.002	0.181 (0.073-0.451)	<0.001

CTP, Child-Pugh Score; ALBI, albumin-bilirubin; AFP, alpha-fetoprotein; PIVKA, proteins induced by vitamin K absence or antagonism-II; HBV, hepatitis B virus; BCLC, Barcelona Clinic Liver Cancer

Table S4. Contingency table depicting the association of the KUSS50 with the benefit of atezolizumab treatment in the GO30140 cohort.

Prediction	Partial response or better	Non-responder	Total
Benefit	5	12	17
Non-benefit	4	22	26
Total	9	34	43

p = 0.48 by χ^2 test

Table S5. Two-way contingency table depicting the association of the KUSS50 with the benefit of atezolizumab and bevacizumab treatment in the IMbrave150 + GO30140 cohort.

Prediction	Partial response or better	Non-responder	Total
Benefit	28	56	84
Non-benefit	53	110	163
Total	81	166	247

p = 0.91 by χ^2 test

Table S6. Activated upstream regulators in the non-benefit subtype predicted by gene network analysis.

Upstream regulator	Molecular feature	Predicted state	Activation *z*-score	*p*-value	Genes
**MYC**	Transcription regulator	Activated	5.789	0.000831	64
**CTNNB1**	Transcription regulator	Activated	4.56	1.93E-13	59
**HNF1A**	Transcription regulator	Activated	4.196	6.22E-14	52
**HGF**	Growth factor	Activated	4.09	0.00144	28
**N6AMT1**	Enzyme	Activated	4.082	4.03E-06	17
**STAT3**	Transcription regulator	Activated	4.068	0.0139	37
**SP1**	Transcription regulator	Activated	4.053	0.000662	34
**RELA**	Transcription regulator	Activated	4.032	0.00701	26
**CD40**	Transmembrane receptor	Activated	3.908	0.015	26
**MYCN**	Transcription regulator	Activated	3.869	0.000159	24
**NFKB**	Transcription regulator	Activated	3.836	0.0127	30
**MLXIPL**	Transcription regulator	Activated	3.712	0.000202	14
**ERBB2**	Kinase	Activated	3.618	2.27E-07	64
**AHR**	Nuclear receptor	Activated	3.604	6.39E-07	41
**TCF7L2**	Transcription regulator	Activated	3.57	0.000162	28
**NFE2L2**	Transcription regulator	Activated	3.569	0.000053	43
**CD44**	Other	Activated	3.541	0.0045	16
**IL27**	Cytokine	Activated	3.537	0.0115	14
**ERK1/2**	Kinase	Activated	3.414	0.000393	22
**EPO**	Cytokine	Activated	3.372	0.0014	20

This table summarizes the results of the Ingenuity Pathway Analysis (IPA) of genes upregulated in the non-benefit subtype, highlighting predicted upstream regulators that may drive the observed transcriptional profile. Only the top 20 upstream regulators are shown, ranked by activation *z*-score.

Upstream regulators: The top 20 genes or molecules predicted to regulate downstream targets in the dataset.
Molecular features: The functional class of the regulator (transcription factor, kinase, cytokine, etc.).
Predicted state: Indicates whether the upstream regulator is predicted to be activated based on the direction of expression changes in its known targets.
Activation *z*-score: A statistical measure of regulator activity; scores >2 suggest activation with confidence, with higher values indicating stronger predicted activation.
***p*-value:** Statistical significance of the overlap between known targets of the regulator and the differentially expressed genes estimated by the Fisher exact test; lower values indicate stronger enrichment.
Genes: Number of genes in the dataset known to be regulated by the upstream regulator.
