# Supplementary material for: Identifying sorafenib benefit among patients with hepatocellular carcinoma: A transcriptomic and genomic approach
Source: JHEP Rep. 2026 Jan 27;8(4):101742. doi: 10.1016/j.jhepr.2026.101742 (PMC12969672; doi:10.1016/j.jhepr.2026.101742)
Supplement: Multimedia component 4 [file mmc4.pdf]

Research article

JHEP|Reports

Identifying sorafenib benefit among patients with hepatocellular carcinoma: A transcriptomic and genomic approach

Authors

Sun Young Yim, Hayeon Kim, Tae Hyung Kim, …, Han Liang, Ju-Seog Lee, Ji Hoon Kim

Correspondence

jlee@mdanderson.org (J.-S. Lee), kjhhepar@naver.com, kjhhepar@korea.ac.kr (J.H. Kim).

Graphical abstract

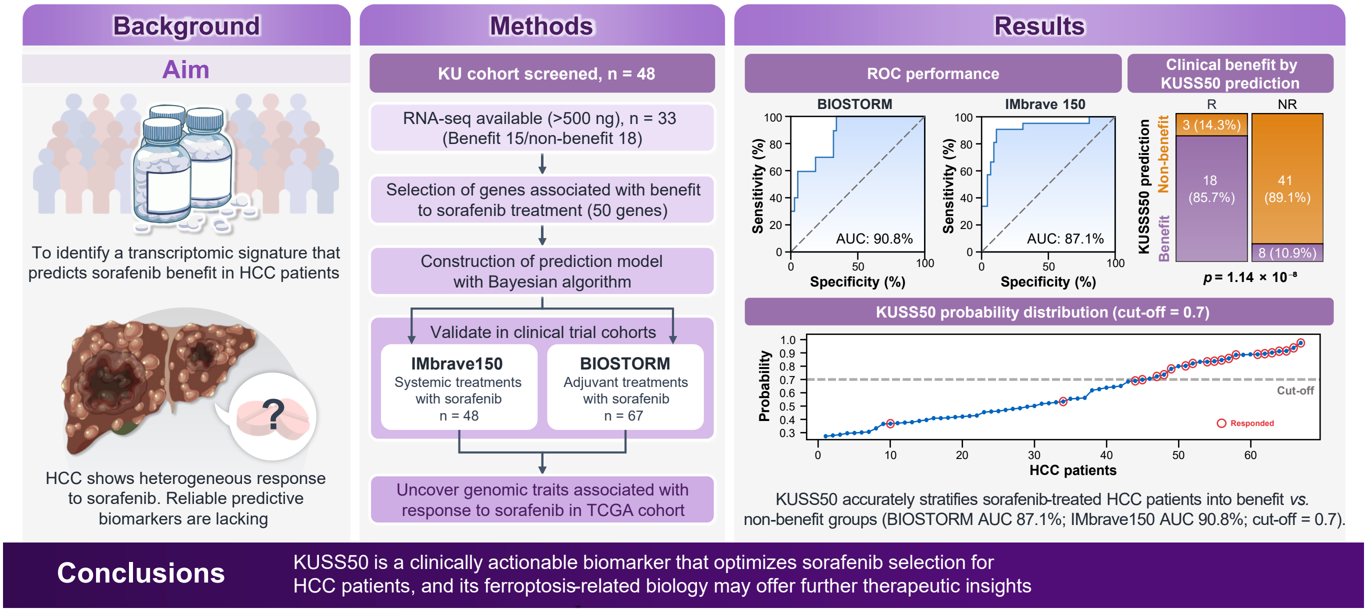

Highlights:

- The KUSS50 is a novel 50-gene signature predicting the benefit of sorafenib treatment in HCC.
- High predictive accuracy was validated across multiple independent cohorts.
- KUSS50-defined subtypes have distinct molecular characteristics linked to treatment outcomes.
- The KUSS50 signature enables personalized sorafenib therapy, improving patient selection.
- Insights offer new therapeutic avenues for enhancing HCC treatment efficacy.

Impact and implications:

This study identifies the KUSS50, a novel 50-gene signature, as a predictive biomarker for identifying patients with hepatocellular carcinoma (HCC) who are likely to benefit from sorafenib treatment. The findings have significant implications for the clinical management of HCC, particularly in optimizing treatment strategies and enhancing patient outcomes. The ability to predict the benefit of sorafenib treatment with high specificity allows for more personalized therapy, reducing unnecessary exposure to ineffective treatments. This approach can be directly applied by clinicians to improve treatment selection, ultimately leading to better patient outcomes. Additionally, understanding the molecular mechanisms underlying the KUSS50-defined subtypes may pave the way for new therapeutic strategies and interventions aimed at improving the efficacy of sorafenib and other treatments in patients with HCC.

https://doi.org/10.1016/j.jhepr.2026.101742
© 2026 The Authors. Published by Elsevier B.V. on behalf of European Association for the Study of the Liver (EASL). This is an open access article under the CC BY license (http://creativecommons.org/licenses/by/4.0/). JHEP Reports, 2026, 8, 1–12

Identifying sorafenib benefit among patients with hepatocellular carcinoma: A transcriptomic and genomic approach

Sun Young Yim[1], Hayeon Kim[2], Tae Hyung Kim[3], Sang-Hee Kang[4], Youngwoo Lee[1], Eunho Choi[1], Yang Jae Yoo[1], Seong Hee Kang[1], Young-Sun Lee[1], Young Kul Jung[1], Yeon Seok Seo[1], Hyung Joon Yim[1], Jong Eun Yeon[1], Kyung Suk Yang[5], Yitao Tang[6], Bowha Sohn[7], Yun Seong Jeong[7], Hyewon Park[7], Han Liang[7], Ju-Seog Lee[7,*], Ji Hoon Kim[1,*]

JHEP Reports **2026**. vol. 8 | 1–12

Check for updates

Background & Aims: Sorafenib has been a cornerstone of hepatocellular carcinoma (HCC) therapy; however, its efficacy is limited, and identifying patients who will benefit from sorafenib is challenging. We aimed to identify predictive biomarkers of sorafenib benefit in patients with HCC.

Methods: Gene expression data from 33 HCC tumors treated with sorafenib were analyzed to construct a prediction model aimed at identifying patients with greater benefit from sorafenib treatment. The robustness of the predictor was validated using gene expression data from two phase III clinical trials, IMbrave150 and STORM.

Results: The analysis of transcriptome data revealed a 50-gene signature, the KUSS50 (Korea University Sorafenib Signature with 50 genes), that exhibited high predictive power in identifying patients who benefited from sorafenib treatment in a training cohort. Validation in two independent cohorts – IMbrave150 (n = 48) and BIOSTORM (n = 67) –demonstrated high specificity for predicting sorafenib benefit (AUC: 87.1%, $p = 1.8 \times 10^{-4}$ and 90.8%, $p = 1.0 \times 10^{-7}$, respectively). Genomic analyses identified distinct molecular characteristics associated with the KUSS50-defined benefit subtype, including an increased mutation rate and activation of ferroptosis, suggesting increased baseline ferroptotic activity in these HCCs, which may sensitize them to sorafenib. The benefit subtype also overlapped with previously defined HCC subtypes associated with stemness and aggressiveness. Conversely, the non-benefit subtype correlated with β-catenin mutations and increased tumor purity, underscoring its biological significance.

Conclusions: The KUSS50 is a clinically actionable biomarker that may optimize patient selection for sorafenib treatment in HCC, potentially improving outcomes. Further exploration of the underlying biology of KUSS50-defined subtypes – particularly the role of ferroptosis in sorafenib sensitivity – may yield additional therapeutic insights.

© 2026 The Authors. Published by Elsevier B.V. on behalf of European Association for the Study of the Liver (EASL). This is an open access article under the CC BY license (http://creativecommons.org/licenses/by/4.0/).

Introduction

Hepatocellular carcinoma (HCC) is a highly aggressive and lethal liver cancer, accounting for a notable portion of cancer-related deaths worldwide.[1,2] HCC primarily affects individuals with chronic liver diseases, such as cirrhosis, hepatitis B or C virus infection, and metabolic dysfunction–associated steatotic liver disease.[3–6] Despite advances in diagnostic techniques and treatment strategies, HCC remains a significant global health burden, with a 5-year survival rate of only 18%.[7]

Sorafenib, a multikinase inhibitor, has been the cornerstone of systemic therapy for advanced HCC since its approval by the US FDA in 2007.[8,9] Sorafenib primarily targets the serine-threonine kinases Raf-1 and B-Raf as well as the receptor tyrosine kinases vascular endothelial growth factor receptor and platelet-derived growth factor receptor.[10] By inhibiting these kinases, sorafenib restrains tumor cell proliferation, angiogenesis, and tumor cell survival, ultimately leading to improved survival for patients with advanced HCC. Despite the initial success of sorafenib in HCC treatment, its long-term efficacy remains limited owing to several factors, including intrinsic and acquired resistance, tumor heterogeneity, and the complex tumor microenvironment.[11–14] Additionally, sorafenib is associated with various adverse effects, including fatigue, diarrhea, hand and foot skin reactions, and hypertension, which notably reduce patients' quality of life and adherence to treatment.[12] Furthermore, not all patients derive equal benefit from sorafenib therapy,[8,9,11,12] underscoring the need for reliable biomarkers to predict treatment outcomes.

To address these limitations and improve patient outcomes, several studies have identified predictive biomarkers to guide

* Corresponding author. Addresses: Department of Systems Biology, Unit 1058, The University of Texas MD Anderson Cancer Center, 1515 Holcombe Blvd., Houston, TX 77030, USA. Tel.: +1 713 834 6154, fax: +1 713 563 4235; (J.-S. Lee), or Department of Internal Medicine, Korea University Medical Center, 148 Gurodong-ro, Guro-gu, Seoul 08308, South Korea. Tel.: +82-2-2626-1031, fax: +82-2-2626-1038 (J.H. Kim).
E-mail addresses: jlee@mdanderson.org (J.-S. Lee), kjhhepar@naver.com, kjhhepar@korea.ac.kr (J.H. Kim).
https://doi.org/10.1016/j.jhepr.2026.101742

patient selection and optimize sorafenib use. For instance, Ang-2 was identified as a potential biomarker of poor outcome for patients with HCC given sorafenib.[15] Furthermore, *VEGFA* amplification in HCC cells was associated with better outcomes for patients who underwent sorafenib treatment.[16] Additionally, polymorphisms in genes involved in sorafenib metabolism, such as polymorphisms relevant to UDP-glucuronosyltransferase 1A9, have been linked with sorafenib response and toxicity.[17]

Although several biomarkers for predicting sorafenib treatment benefit have been proposed, their clinical utility and reproducibility remain subject to validation in large multicenter cohorts. Challenges such as interpatient variability, tumor heterogeneity, and the dynamic nature of treatment response underscore the need for robust and reliable biomarkers that can accurately stratify patients with HCC as those who would and those who would not benefit from sorafenib treatment, guiding treatment decisions. Moreover, integrating multiple biomarkers into predictive models or nomograms may bolster the predictive precision and clinical usefulness of such models and nomograms, providing clinicians with valuable tools for treatment selection and monitoring. In the present investigation, we aimed to address these critical needs by analyzing the transcriptome data of HCC samples collected during prospective clinical trials. Using an in-depth analysis, we sought to identify predictive biomarkers essential for optimizing sorafenib treatment strategies and ultimately improving patient outcomes.

Patients and methods

Patients, sample collection, and RNA sequencing

Demographic information, clinical data, and tissue samples were obtained for 33 patients who had undergone sorafenib treatment from 2010 to 2018 at Korea University (KU) Hospital (Table S1), under the approval of the Institutional Review Board (IRB No. 2017GR0176 and 2018AN0445). Most of the patients were male (81.8%), had hepatitis B virus infection (72.7%), and presented with extrahepatic metastasis (90.9%) at the initiation of sorafenib. Among the patients whose disease progressed during sorafenib treatment, 42.4% received second-line chemotherapy. The median follow-up duration was 12.6 months (range, 1.3-69.5 months).

All patients underwent liver biopsy analysis prior to sorafenib use, and total RNA was obtained from their formalin-fixed, paraffin-embedded tissue blocks. Total RNA was extracted from slides containing tissue sections from these blocks using a Purigen Biosystems Ionic Purification System, and the DV200 values (representing the percentage of fragments >200 nucleotides) exceeded 15%. mRNA expression data for HCCs in the study patients were subsequently generated on the Illumina NovaSeq 6000 platform, employing S4-xp-200 lanes with a sequencing depth of 50 million reads per sample. The quality of the raw reads was checked using FastQC (v0.11.5) and summarized using MultiQC (v1.7). FASTQ files were mapped to the human reference genome (GRCh38) using STAR (v2.7.10a), and gene expression levels were quantified using RSEM (v1.3.3) with default parameter settings. The resulting transcriptomic dataset is available in the NCBI Gene Expression Omnibus under accession number GSE285625.

Gene expression and clinical data from validation cohorts

Gene expression and clinical data from two clinical trials (IMbrave150 and GO30140) were obtained from Genentech, and all datasets are available from the European Genome-Phenome Archive database (accession number EGAD00001008128).[18–20] The IMbrave150 (NCT03434379) trial was a phase III clinical study evaluating the safety and efficacy of atezolizumab plus bevacizumab *vs.* sorafenib as the primary treatment for unresectable HCC.[18,21] Similarly, in the GO30140 (NCT02715531) trial, researchers investigated the safety and dosage of atezolizumab alone and combined with bevacizumab for patients with HCC.[19] The key endpoints of these studies included the confirmed objective response rate in all patients who received the treatments and overall survival and progression-free survival in the intention-to-treat population, which were assessed using an impulse residue function according to RECIST v1.1.[22] Combining the patient populations from both trials resulted in 253 patients in the atezolizumab-plus-bevacizumab arm, 47 patients in the atezolizumab-only arm, and 58 patients in the sorafenib arm with available gene expression data. Patients without clinical outcome data were excluded, leaving 247 patients in the atezolizumab-plus-bevacizumab arm, 43 patients in the atezolizumab-only arm, and 48 patients in the sorafenib arm for analysis.

Gene expression and clinical data from the BIOSTORM trial cohort were obtained from the Gene Expression Omnibus under accession number GSE109211.[23,24] The BIOSTORM cohort was a subset of the cohort in the phase III STORM trial focused on biomarker research. The National Cancer Institute proliferation (NCIP) subtype was used as an exemplar genomic subtype significantly associated with HCC prognosis, as previously described.[25–27] Additionally, genomic, histological, and clinical data – including information on mutations and copy number alterations – from The Cancer Genome Atlas (TCGA) HCC project were obtained and analyzed in accordance with prior studies.[28–31]

Data analysis

Collected gene expression data were transformed and normalized as described previously.[25–31] BRB-ArrayTools (v4.6.1), a freeware program from the National Cancer Institute (http://brb.nci.nih.gov/BRB-ArrayTools.html), was used to analyze the data and build a predictive model.[32] Cluster and TreeView were used to generate a heat map of gene expression data.[33] All statistical analyses were performed in the R language (http://www.r-project.org).

To identify genes associated with the benefit of systemic sorafenib administration in patients with HCC, we selected genes whose expression was significantly different (p <0.05 [Student's t test]) between patients in a KU training cohort who did (benefit group) and did not (non-benefit group) experience treatment benefit. The benefit group was defined as patients with controlled disease, which included objective response and stable disease evaluated according to RECIST v1.1. following sorafenib treatment, whereas the non-benefit group was defined as patients with progressive disease. To construct a Bayesian prediction model, we selected 50 genes whose expression was most significantly upregulated in the benefit group. The resulting 50-gene signature was named the KU Sorafenib Signature with 50 genes (KUSS50).

Before independent gene expression data were pooled to perform each analysis, each gene expression dataset was independently standardized to have a mean of 0 and standard deviation of 1 as described previously.[25–31] To estimate the likelihood that a specific human HCC would exhibit a particular gene expression pattern, we employed a Bayesian compound covariate prediction algorithm based on training sets. The gene expression data from the KU cohort, which served as the training sets, were combined to construct a predictive model using the Bayesian compound covariate prediction framework. To assess the model's robustness, we conducted leave-one-out-cross-validation during the training process. A Bayesian probability threshold of 0.7 yielded the highest positive prediction value of 0.917 and a negative prediction value of 0.81.

Receiver-operating characteristic (ROC) curve analyses were carried out to estimate the discriminatory power of the KUSS50. The AUC was calculated, which ranged from 0.5 (for a non-informative predictive marker) to 1 (for a perfect predictive marker), and a bootstrap method (1,000 resampling) was used to calculate the 95% CI for the AUC. Multivariate analysis using Cox regression was performed for overall survival to identify factors that could predict an overall survival benefit from sorafenib.

Tumor purity in TCGA samples was assessed using three complementary approaches. Tumor purity was defined as the proportion of tumor cells relative to the total cell population within a given sample. First, histopathological evaluation was performed using conventional H&E staining. Board-certified pathologists at the Biospecimen Core Resource of Nationwide Children's Hospital reviewed digital H&E-stained slides and visually estimated tumor cellularity in representative regions with minimal necrosis or hemorrhage.[31] Second, we applied the ESTIMATE algorithm, which infers stromal and immune cell infiltration based on the gene expression profiles of 141 immune-related and 141 stromal-related genes.[34] In this RNA-based method, higher stromal or immune scores reflect greater non–tumor cell content, and tumor purity was calculated as one minus the sum of the normalized stromal and immune scores. Third, tumor purity was estimated using ABSOLUTE, a DNA-based method that models allele-specific somatic copy-number alterations and heterozygosity.[35] This approach provided a model-derived estimate of tumor purity for each TCGA sample.

Results

Gene expression signature associated with benefit from sorafenib treatment

To identify potential biomarkers that could predict which patients would benefit from treatment with sorafenib, we categorized patients in the KU cohort into two groups based on their responses to the drug. The non-benefit group consisted of 18 patients who received sorafenib and experienced disease progression, whereas the benefit group consisted of 15 patients who received sorafenib and did not experience disease progression. For all patients, the median overall survival duration was 12.6 months. As expected, there was a statistically significant difference in overall survival between the benefit and non-benefit groups (Fig. S1). We sought to identify genes whose expression levels differed significantly (p <0.05 [Student's t test]) between the benefit and non-benefit groups. Of these differentially expressed genes, we focused on those that exhibited higher expression in the benefit group than in the non-benefit group. This strategic selection of upregulated genes in the benefit group was intended to facilitate the development and validation of a predictive model for independent patient cohorts (Fig. S2). Through this approach, we identified the 50 most upregulated genes in the benefit group as candidate biomarkers for predicting sorafenib benefit (Fig. 1A and S3, Table S2). We defined this set of 50 genes as the KUSS50. We then used the KUSS50 to construct a Bayesian prediction model aimed at identifying patients with HCC who would potentially benefit from sorafenib treatment (Fig. 1B). To assess the robustness of the KUSS50 in predicting sorafenib benefit within the KU training cohort, we performed ROC analysis. The AUC for the Bayesian probability derived from the KUSS50 model was remarkably high at 92.6% (95% CI 82.2-99.3%; $p = 2.6 \times 10^{-6}$), demonstrating the signature's significant ability to discriminate between the benefit and non-benefit patients in this cohort (Fig. 1C). Multivariate analysis using Cox regression was performed to identify independent factors associated with benefit from sorafenib and revealed that the KUSS50, along with AFP level and patient performance status, remained significantly associated with sorafenib benefit (Table S3).

Validation of the KUSS50 in the IMbrave150 first-line treatment cohort

After identifying that the KUSS50 was significantly associated with favorable outcomes in patients with HCC receiving systemic sorafenib therapy, we attempted to validate the predictive capability of the KUSS50-based Bayesian probability model in an independent cohort. We carried out this validation using the gene expression data of the HCC tissues of patients enrolled in the sorafenib arm of the phase III IMbrave150 clinical trial.[18,20] We evaluated the predictive performance of the KUSS50-based Bayesian probability model by assessing its ability to accurately identify patients who benefited from sorafenib use, defined as those who had at least a partial response. Remarkably, the AUC calculated in the ROC analysis of the IMbrave150 sorafenib arm was 87.1% (95% CI 75.8-96.1%; $p = 1.8 \times 10^{-4}$) (Fig. 2A). This AUC value was similar to that for the original KU training cohort, underscoring the robustness of the KUSS50 model. To further validate the KUSS50's predictive power, we stratified patients in the IMbrave150 sorafenib arm based on a predefined Bayesian probability cut-off of 0.7 (Fig. 2B). This stratification revealed a significant statistical association (p = 0.003 [chi-square test]) between the KUSS50-predicted subtypes and clinical benefit of sorafenib use (Table 1; Fig. 2C). Patients sorted into the benefit group by the KUSS50 model exhibited a substantially higher rate of achieving at least a partial response than those sorted into the non-benefit group.

Notably, when applied to the atezolizumab-only or atezolizumab-plus-bevacizumab treatment arms in the IMbrave150 trial, the KUSS50-predicted subtypes were not significantly associated with treatment benefit (Tables S4 and S5). This observation underscores the high specificity of the KUSS50, which appears to predict the benefit of sorafenib, but not that of other therapeutic regimens, in HCC. This validation in the independent IMbrave150 cohort not only confirmed the

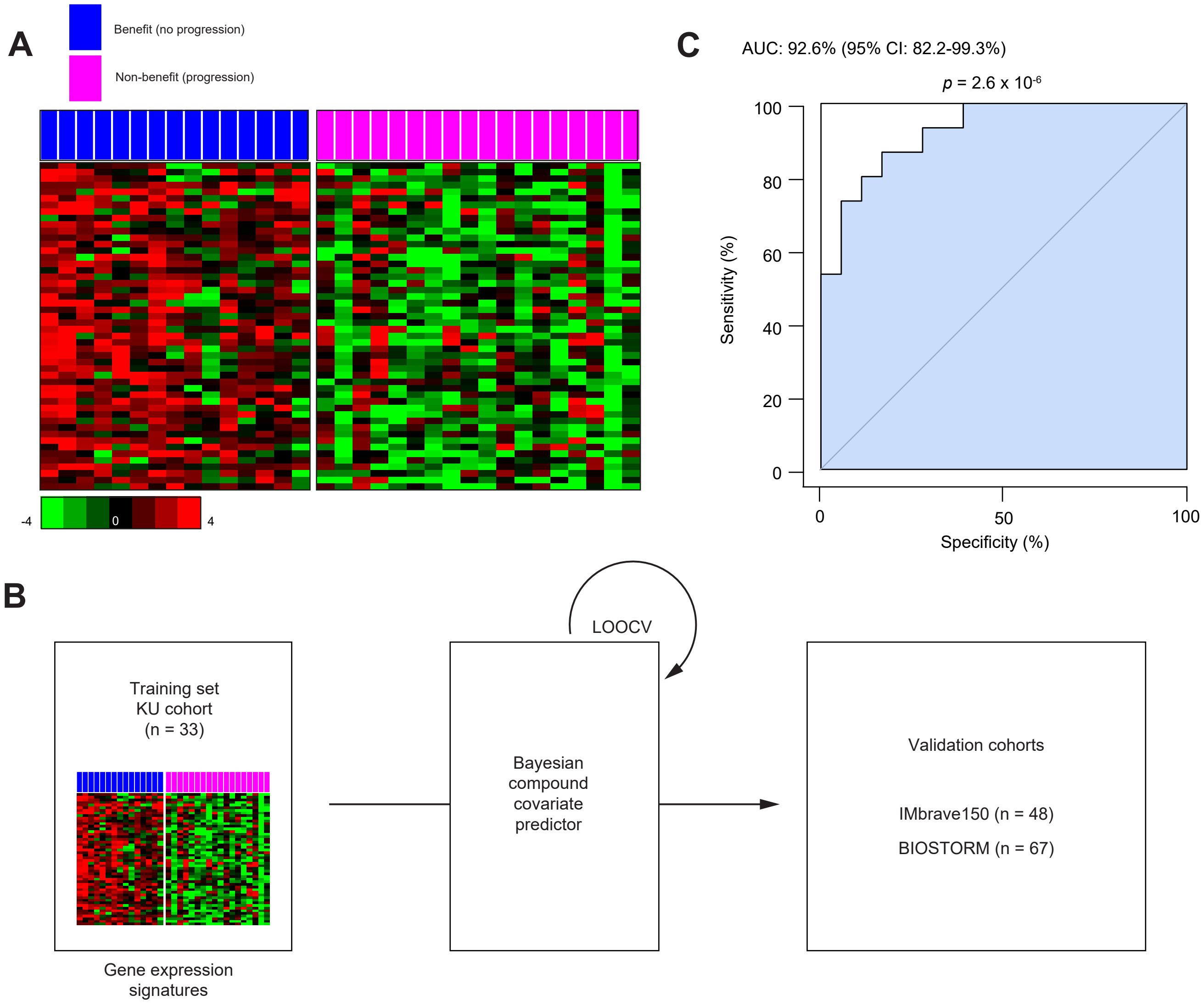

Fig. 1. Genes associated with a benefit of sorafenib treatment in patients with HCC, and the development of a KUSS50 prediction model. (A) Heatmap showing the expression patterns of the 50 genes constituting the KUSS50. Genes were identified in the KU training cohort based on their differential expression between the benefit and non-benefit groups using the Student's *t* test (p <0.05) and ranked by fold-change (benefit/non-benefit). Bars indicate relative expression (*z*-score). (B) Schematic illustration of the Bayesian compound covariate predictor model built with the KUSS50 gene set. The model was trained with the KU cohort (n = 33) and validated with two independent phase III clinical trial datasets (IMbrave150, n = 48; BIOSTORM, n = 67) using LOOCV. (C) Receiver-operating characteristic curve showing the performance of the Bayesian predictor probability for distinguishing patients with HCC who benefited from sorafenib treatment in the KU training cohort during LOOCV. The AUC was 0.926 (95% CI 0.822–0.993), with statistical significance assessed by a bootstrap test with 1,000 resamplings ($p = 2.6 \times 10^{-6}$). The AUC and 95% CI were computed using the pROC package (bootstrap method). HCC, hepatocellular carcinoma; KU, Korea University; KUSS50, Korea University Sorafenib Signature with 50 genes; LOOCV, leave-one-out cross-validation.

predictive capability of the KUSS50 model but also highlighted the signature's remarkable specificity for sorafenib benefit, further reinforcing its potential clinical use in optimizing patient selection for the targeted therapy.

Validation of the KUSS50 in the BIOSTORM adjuvant treatment cohort

Encouraged by the compelling correlation observed between the KUSS50 predictor and positive treatment outcomes in patients receiving first-line sorafenib therapy for HCC, we aimed to expand the validation process by determining whether the KUSS50 predictor could effectively identify patients who would benefit from receiving sorafenib in the adjuvant setting following primary treatment. To accomplish this, we used gene expression data from the BIOSTORM cohort. The BIOSTORM cohort is a subset of patients from the phase III STORM trial, which investigated potential biomarkers in patients with HCC receiving adjuvant therapy.[23,24]

We applied the KUSS50-based model to the gene expression data from the BIOSTORM cohort and evaluated its ability to accurately identify patients who benefited from adjuvant sorafenib use (defined as responders [recurrence-free patients] in the BIOSTORM study). Remarkably, the AUC from the ROC analysis was 90.8% (95% CI 81.5-97.5%; $p = 1.0 \times 10^{-7}$) (Fig. 3A). This AUC value closely mirrored those for both the

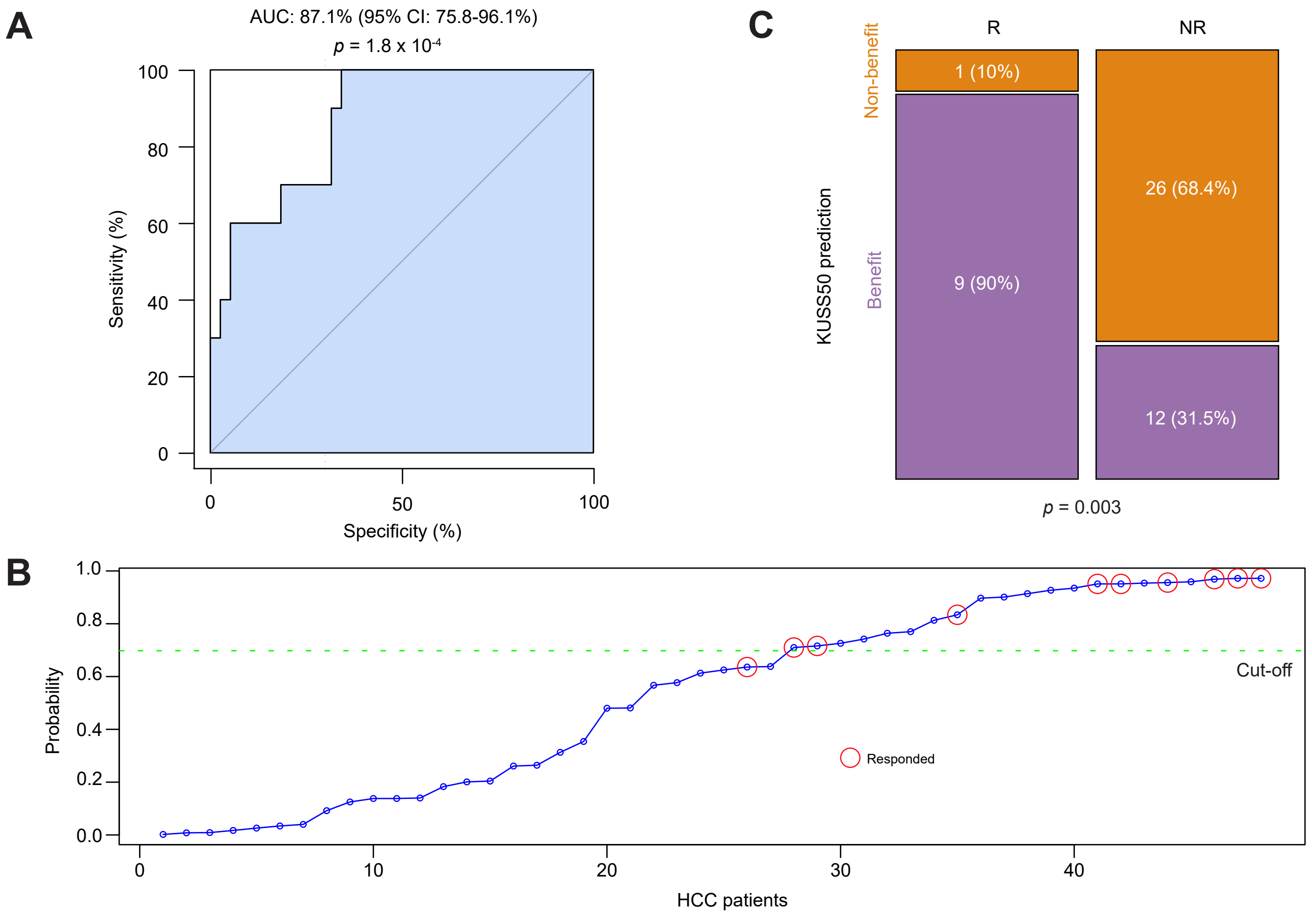

Fig. 2. Significant correlation between the KUSS50 and improved outcomes in patients with HCC who underwent sorafenib treatment in the IMbrave150 trial cohort. (A) Receiver-operating characteristic curve illustrating the performance of the KUSS50-based Bayesian probability model for identifying patients who benefited from sorafenib treatment (*i.e.* who achieved at least a partial response). The AUC was 0.871 (95% CI 0.758–0.961), and statistical significance was determined using a bootstrap test with 1,000 resamplings ($p = 1.8 \times 10^{-4}$). (B) Distribution of the KUSS50 Bayesian probability scores for each patient in the IMbrave150 cohort. The stratification cut-off of 0.7 was predetermined from LOOCV in the KU cohort and used to separate predicted responders (benefit) from non-responders (no benefit). (C) Stacked bar plot showing the proportions of patients in each response category predicted by the KUSS50-based model. Among the 10 predicted responders, 9 (90%) achieved clinical benefit, whereas among the 38 predicted non-responders, 12 (31.5%) did ($p = 0.003$; Fisher exact test). HCC, hepatocellular carcinoma; KU, Korea University; KUSS50, Korea University Sorafenib Signature with 50 genes; LOOCV, leave-one-out cross-validation; NR, no response; R, responded to treatment by achieving at least a partial response.

original KU training cohort and the IMbrave150 sorafenib-only cohort, further validating the robustness of the KUSS50 predictor. To corroborate the predictive power of the KUSS50 model, we stratified patients in the BIOSTORM cohort based on a predefined Bayesian probability cut-off of 0.7 (Fig. 3B). This stratification revealed a highly significant association ($p = 1.14 \times 10^{-8}$ [chi-square test]) between the KUSS50 prediction and the clinical benefit derived from adjuvant sorafenib treatment (Table 2; Fig. 3C). Patients assigned to the benefit group by the KUSS50 model exhibited substantially better outcomes than those in the non-benefit group.

Table 1. Contingency table depicting the significant association of the KUSS50-based model with sorafenib benefit in the IMbrave150 cohort.

Prediction	Responder (Partial response or better)	Non-responder	Total
Benefit	9	12	21
Non-benefit	1	26	27
Total	10	38	48

$p = 0.003$ by χ^2 test.
KUSS50, Korea University Sorafenib Signature with 50 genes.

This finding in the BIOSTORM cohort provided further compelling evidence that the KUSS50 predictor can effectively identify patients with HCC who are likely to benefit from treatment with sorafenib not only in the first-line setting but also in the adjuvant setting following primary treatment. The KUSS50 model performed consistently well across multiple independent cohorts receiving first-line or adjuvant sorafenib, underscoring its potential clinical utility in guiding personalized treatment decisions and optimizing patient selection for sorafenib therapy.

Genomic and histological characteristics associated with KUSS50-defined subtypes

In subsequent analyses, we assessed genomic characteristics associated with KUSS50-defined subtypes in the TCGA cohort to gain further insight into their underlying biology. Initially, we

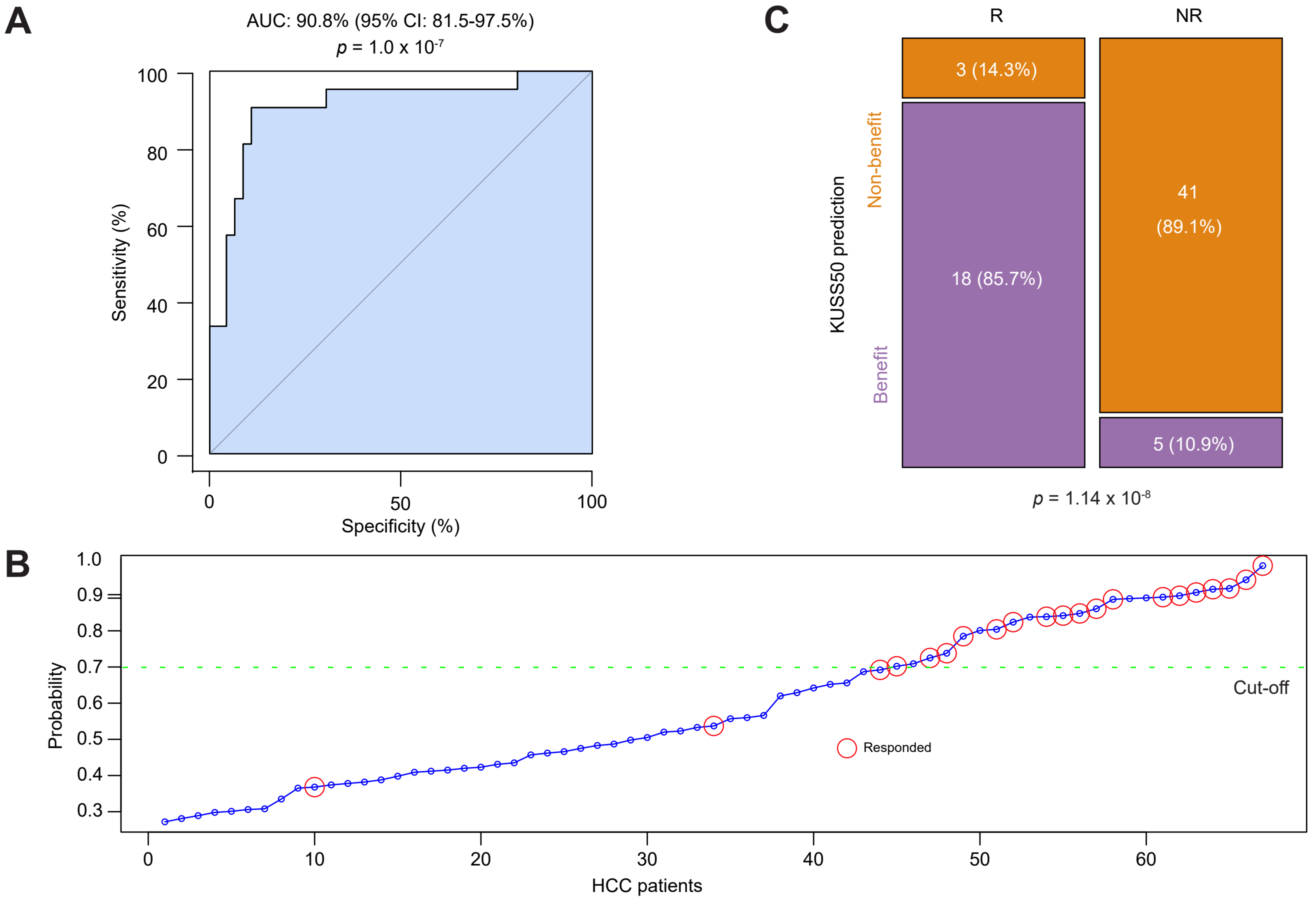

Fig. 3. Significant correlation between the KUSS50 and improved outcomes in patients with HCC who underwent sorafenib treatment in the BIOSTORM trial. (A) Receiver-operating characteristic curve evaluating the performance of the KUSS50-based Bayesian probability model in identifying patients with HCC who benefited from sorafenib treatment in the BIOSTORM trial cohort. The AUC was 0.908 (95% CI 0.815–0.975), and statistical significance was determined using a bootstrap test with 1,000 resamplings ($p = 1.0 \times 10^{-7}$). (B) Distribution of the KUSS50 Bayesian probability scores for each patient in the BIOSTORM cohort. The cut-off probability of 0.7, established during leave-one-out cross-validation in the KU training cohort, was used to stratify patients into predicted benefit and non-benefit groups. (C) Stacked bar plot showing the proportions of patients in each response category predicted by the KUSS50 in the BIOSTORM cohort. Among 21 patients predicted to have a benefit, 18 (85.7%) achieved clinical benefit, whereas of 46 patients predicted to have no benefit, 5 (10.9%) achieved clinical benefit ($p = 1.14 \times 10^{-8}$; Fisher exact test). HCC, hepatocellular carcinoma; KU, Korea University; KUSS50, Korea University Sorafenib Signature with 50 genes; NR, no response; R, responded to treatment by achieving recurrence-free.

compared the genomic copy-number alterations between the two KUSS50-defined subtypes, finding no discernible differences (Fig. 4A). However, we observed a notable difference in the mutation burden between the subtypes, with the non-benefit subtype exhibiting markedly higher mutation rates than its counterpart (Fig. 4B).

We then proceeded to identify somatic mutations that were significantly associated with the KUSS50-defined subtypes (Fig. S4). Whereas mutations of *TP53* exhibited no notable correlations with the subtypes, mutations of *CTNNB1* (encoding β-catenin) and *AXIN1* were notably enriched in the non-benefit subtype. This observation suggests the involvement of the β-catenin pathway in conferring resistance to sorafenib, which is in alignment with prior studies demonstrating that the activation of β-catenin is linked with sorafenib resistance.[36–38] Notably, mutations of the *ALB* and *APOB* genes, which encode plasma proteins, were significantly associated with the non-benefit subtype, suggesting a connection between loss of plasma proteins and resistance to sorafenib. Conversely, mutations of *BAP1* and *FAM47A* were predominantly associated with the benefit subtype.

Furthermore, an analysis of H&E-stained tumor sections from the TCGA cohort demonstrated that the KUSS50-defined non-benefit subtype exhibited significantly higher tumor purity compared to the benefit subtype (Fig. 4C). This observation suggests that reduced stromal cell–cancer cell interactions underlie the limited responsiveness to sorafenib. Consistent with this, genome-based tumor purity estimates derived from

Table 2. Contingency table depicting the significant association of the KUSS50-based model with sorafenib benefit in the BIOSTORM cohort.

Prediction	Responder (recurrence-free)	Non-responder (recurrence)	Total
Benefit	18	5	23
Non-benefit	3	41	44
Total	21	46	67

$p = 1.14 \times 10^{-8}$ by χ^2 test.
KUSS50, Korea University Sorafenib Signature with 50 genes.

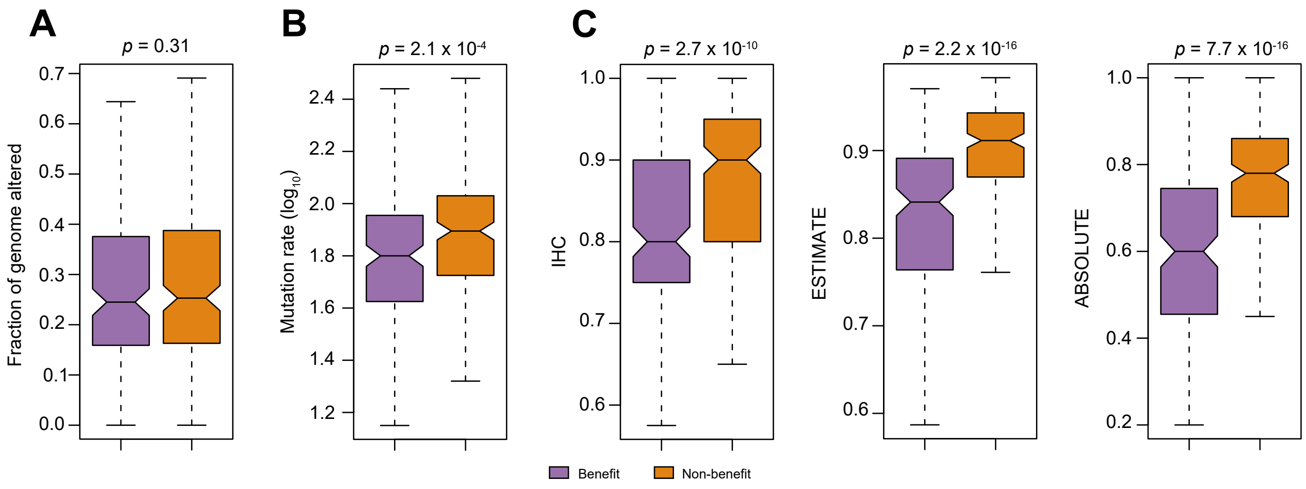

Fig. 4. Genomic alterations and purity scores associated with the KUSS50-defined subtypes. (A) Box plot showing the fraction of the genome altered by copy-number gain and loss in HCC samples from the TCGA-LIHC dataset (n = 371). Differences between the KUSS50-defined benefit and non-benefit groups were assessed using the Student's *t* test ($p = 0.31$). (B) Comparison of non-synonymous mutation burdens between KUSS50-defined subtypes in the TCGA-LIHC cohort (n = 367). The benefit group showed a significantly higher mutation rate than the non-benefit group ($p = 2.1 \times 10^{-4}$, Student's *t* test). (C) Box plots of tumor purity estimated by three independent methods: pathologic assessment of tumor-cell percentages using IHC, computational inference using the ESTIMATE algorithm, and computational inference using the ABSOLUTE algorithm. Tumor purity was significantly higher in the non-benefit group. Differences were assessed using the Student's *t* test. Box boundaries represent the 25th–75th percentiles, center lines indicate the means, and whiskers denote the 10th and 90th percentiles. HCC, hepatocellular carcinoma; IHC, immunohistochemistry; KUSS50, Korea University Sorafenib Signature with 50 genes; TCGA, The Cancer Genome Atlas.

the ESTIMATE (transcriptomic) and ABSOLUTE (copy-number) algorithms showed similar trends.[34,35] As expected, the three independent methods for assessing tumor purity were highly concordant (Fig. S5), further validating the observed differences between the two KUSS50-defined subtypes (Fig. 4C).

Prognostic relevance of KUSS50-defined subtypes and the association with previously identified genomic subtypes

Given the substantial correlation between the KUSS50 and favorable outcomes in patients with HCC undergoing sorafenib treatment that we observed, we next assessed the prognostic significance of the KUSS50-defined subtypes of HCC. We conducted this analysis using data from patients in the TCGA HCC cohort, most of whom did not receive sorafenib.[31] As a point of reference, in the TCGA cohort, we evaluated the prognostic relevance of the HCC subtypes previously identified by the NCIP predictor,[25–27] which have been shown to be associated with prognosis. As expected, the NCIP predictor–identified subtypes were significantly associated with prognosis in the TCGA patients (Fig. 5A). In contrast, the KUSS50-defined subtypes were not associated with prognosis in the TCGA cohort. This observation strongly demonstrates that although the KUSS50 could serve as a predictive marker for identifying patients with HCC who are likely to benefit from sorafenib administration, it lacks prognostic relevance in a general context in which sorafenib is not administered.

Building on this finding, we further explored the potential correlation between sorafenib sensitivity as defined by the KUSS50-defined subtypes and the previously identified genomic subtypes of HCC. Remarkably, the KUSS50-defined benefit subtype showed notable, but not exclusive, association with several well-established genomic subtypes of HCC. For instance, a substantial portion of benefit cases aligned with the iCluster 1 (iC1) group in the TCGA classification (Fig. S6).[31] Similar trends were observed with Hoshida's S1 and S2 subtypes[39] and with the STEM (stem cell) and ECM (extracellular matrix) subtypes defined by the INSERM classification.[40] Among the four INSERM subtypes, the KUSS50-defined benefit subtype had the strongest correlation with the ECM subtype and had a weaker yet significant correlation with the STEM subtype (Fig. S7). The benefit group also showed enrichment in hepatic stem cell–like subtypes (HS1 and HS2).[29] However, this association was not absolute, as KUSS50-defined benefit cases were also distributed across non-stem–like subtypes. These findings suggest that while it has meaningful overlap with existing molecular classifications, the KUSS50 reflects a distinct aspect of sorafenib sensitivity that may span multiple biological programs.

Taken together, these findings highlight a compelling and consistent correlation between the benefit of sorafenib treatment according to the KUSS50 and specific genomic subtypes of HCC. The KUSS50-defined benefit subtype aligns with certain HCC subtypes across diverse genomic classification systems (Fig. 5B). This convergence of evidence reinforces the association between the benefit of sorafenib treatment and the underlying genomic landscape of HCC, suggesting that specific genomic features contribute to the observed sensitivity or resistance of HCC to sorafenib treatment.

Molecular characteristics of the KUSS50 subtypes

To gain insight into the underlying biological processes and molecular mechanisms associated with the KUSS50, we performed a gene network analysis using the KUSS50 genes. Ingenuity Pathway Analysis revealed that the cAMP response element–binding protein and G protein–coupled receptor signaling pathways were among the activated pathways in the benefit subtype (Fig. S8). Notably, the ferroptosis signaling pathway emerged as a highly activated pathway in the benefit

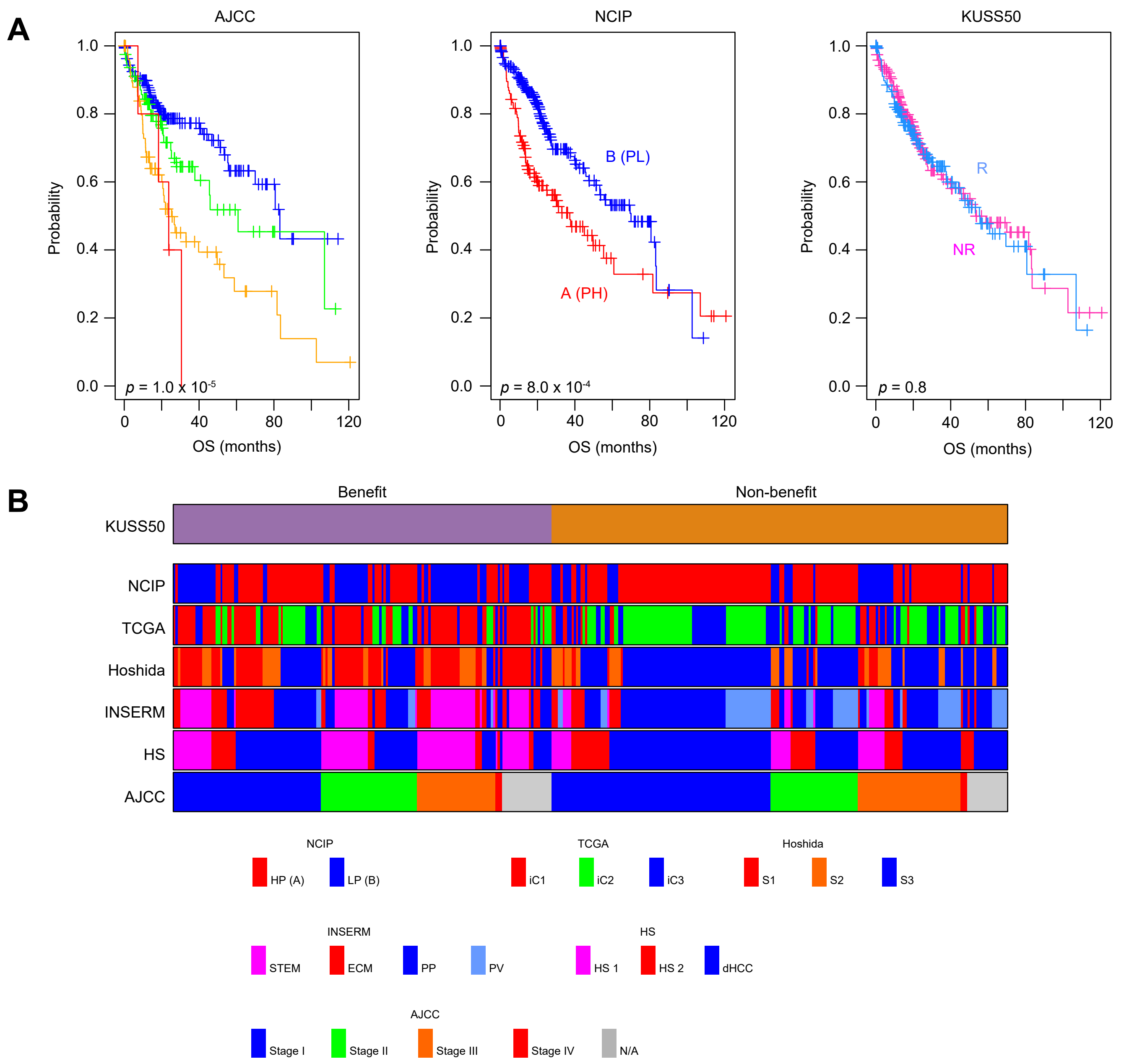

Fig. 5. Association of the KUSS50-defined subtypes with previously identified genomic subtypes of HCC and with HCC prognosis. (A) Kaplan–Meier survival analysis showing OS for patients with HCC from the TCGA-LIHC cohort (n = 365), stratified by AJCC stage, NCIP classification, and the KUSS50 predictor. Survival differences between groups were assessed using the log-rank test. (B) Comprehensive comparative analysis of the KUSS50-defined subtypes with various genomic subtypes of HCC identified and characterized in previous studies. The color map illustrates "the distribution of previously identified subtypes, including NCIP subtypes, TCGA iCluster subtypes, Hoshida subtypes, INSERM subtypes, and hepatic stem cell subtypes, among patients with the KUSS50-defined benefit and non-benefit subtypes. AJCC, American Joint Committee on Cancer; dHCC, differentiated HCC; ECM, extracellular matrix; HCC, hepatocellular carcinoma; HP, high proliferation; HS1, hepatic stem 1; HS2, hepatic stem 2; iC1, iCluster1; iC2, iCluster2; iC3, iCluster3; KUSS50, Korea University Sorafenib Signature with 50 genes; LP, low proliferation; NCIP, National Cancer Institute proliferation; ROC, receiver-operating characteristic; OS, overall survival; PP, periportal; PV, perivenous; STEM, stem cell; TCGA, The Cancer Genome Atlas.

subtype. Ferroptosis is a distinct form of regulated cell death characterized by the iron-dependent accumulation of lipid peroxides in cells.[41,42] The activation of this pathway in the benefit subtype suggested that the increased baseline ferroptotic activity in HCC cells increased their sensitivity to the cytotoxic effects of sorafenib. Supporting this hypothesis, we found that *ACSL4*, a key metabolic gene that promotes ferroptosis, was significantly upregulated in the benefit subtype (Table S2; Fig. S3). *ACSL4* plays a crucial role in the metabolism of polyunsaturated fatty acids,[43] which are particularly susceptible to lipid peroxidation and are necessary for ferroptosis execution.[44,45] The upregulation of *ACSL4* in the benefit subtype suggests that elevated polyunsaturated fatty acid metabolism and increased susceptibility to ferroptosis

contribute to the observed sensitivity of HCC to sorafenib treatment. In addition to the ferroptosis pathway, Ingenuity Pathway Analysis identified the stathmin-1 and phagosome formation pathways as being predicted to be activated in the benefit group. Stathmin-1 is a microtubule-destabilizing protein involved in the regulation of cellular processes such as cell cycle progression and migration.[46]

To investigate the biology underlying the non-benefit subtype, we performed a complementary upstream regulator analysis of the genes upregulated in this group (Table S6). This analysis identified CTNNB1 (β-catenin) as one of the most significantly activated upstream regulators (Fig. S9), consistent with our earlier finding of an increased frequency of activating *CTNNB1* mutations in the non-benefit subtype (Fig. S4). These data strongly support the role of β-catenin signaling in promoting resistance to sorafenib. Beyond *CTNNB1*, other predicted activated upstream regulators included HGF (a ligand for MET), STAT3, and ERBB2 – factors commonly linked to growth factor signaling, survival, and treatment resistance.

These analyses provide important mechanistic insights into the molecular divergence between the KUSS50-defined subtypes. The benefit subtype is characterized by ferroptosis activation and metabolic vulnerability, which may sensitize tumors to sorafenib. In contrast, the non-benefit subtype displays the enrichment of β-catenin signaling and other pro-survival pathways, which may underlie intrinsic resistance. Together, these findings highlight distinct therapeutic vulnerabilities and offer a framework for stratifying patients with HCC based on underlying tumor biology to improve treatment outcomes.

Discussion

In the present study, we conducted a comprehensive analysis of the gene expression data of HCCs in patients who underwent sorafenib treatment. Our analysis led to the identification of the robust 50-gene expression signature, termed the KUSS50, which can accurately identify which patients with HCC are likely to benefit from sorafenib use. The predictive power of the KUSS50 was consistently validated across multiple independent cohorts, highlighting its potential clinical utility in guiding treatment decisions and optimizing patient selection for sorafenib therapy for HCC.

We extensively validated the predictive accuracy of the KUSS50 across two independent cohorts from two phase III clinical trials in which patient data were prospectively collected and analyzed. Notably, the KUSS50 demonstrated high specificity in predicting sorafenib benefit, as demonstrated by several lines of evidence. First, in the IMbrave150 cohort, the Bayesian probability of the KUSS50 exhibited a strong association with clinical outcomes in patients receiving sorafenib but not in those receiving atezolizumab or atezolizumab plus bevacizumab. Second, in the TCGA cohort, in which most patients did not receive sorafenib, the KUSS50 was not associated with prognosis. These findings strongly demonstrate that the KUSS50 can serve as a predictive marker specifically for sorafenib benefit in HCC but lacks prognostic relevance in a general context or predictive power for other treatments. This distinction is crucial for guiding treatment decisions and optimizing patient care, particularly in the context of advanced HCC, for which treatment options are limited.

Our dissection of the molecular characteristics of the KUSS50-defined HCC subtypes produced several intriguing insights. Whereas we observed no significant differences in copy-number alterations between the benefit and non-benefit subtypes, the non-benefit subtype had a higher mutational burden. Notably, mutations of *CTNNB1* and *AXIN1*, components of the Wnt pathway, were enriched in the non-benefit subtype, corroborating previous reports that implicated WNT/β-catenin activation in sorafenib resistance of HCC.[36–38] Conversely, *BAP1* and *FAM47A* mutations were associated with the benefit subtype, warranting further investigation of their potential roles in modulating sorafenib sensitivity in HCC. Another intriguing finding emerged from the analysis of tumor purity, in which the non-benefit subtype exhibited higher cancer cell purity than the benefit subtype, demonstrating that a stromal cell–cancer cell interaction may influence sorafenib benefit. This observation aligns with the well-documented role of the tumor microenvironment in drug resistance and highlights the potential utility of incorporating tumor purity assessments into models designed to predict sorafenib benefit in HCC.[47]

Of note, the KUSS50-defined benefit subtype exhibited striking overlap with previously identified genomic subtypes of HCC, such as the iCluster1 subtype from the TCGA, the S1 and S2 subtypes from Hoshida's classification of HCC, and the STEM and ECM subtypes from the INSERM classification, providing further evidence of the biological relevance of KUSS50 in the context of HCC heterogeneity. The convergence of sorafenib sensitivity with specific genomic subtypes of HCC underscores the importance of integrating molecular profiling into clinical practice to tailor treatment strategies to individual patient profiles.

A pathway analysis of the genes in the KUSS50 revealed the activation of several intriguing pathways in the benefit subtype, including the cAMP response element–binding protein, G protein–coupled receptor, stathmin-1, and phagosome formation pathways. Notably, the ferroptosis signaling pathway was highly activated in the benefit subtype. Ferroptosis, a non-apoptotic form of cell death, is characterized by the iron-dependent accumulation of lipid hydroperoxides in the cell membrane to lethal levels.[41,42] Among the lipid species in the cell membrane, polyunsaturated fatty acids are substrates that are particularly susceptible to lipid peroxidation, and the inhibition of polyunsaturated fatty acid synthesis has been shown to prevent ferroptosis.[44,45]

Notably, *ACSL4*, a gene that plays a crucial role in processing polyunsaturated fatty acids for membrane incorporation and is necessary for promoting ferroptosis,[43] was significantly upregulated in the benefit subtype. This observation aligns with a recent study that demonstrated that the depletion of *ACSL4* makes HCC cells resistant to sorafenib,[48] and it further supports the hypothesis that elevated ferroptotic activity sensitizes cells to sorafenib-based treatment. Previous studies suggested that exposure to sorafenib can induce ferroptosis by inhibiting the SLC7A11/xCT transporter system,[49,50] which is essential for intracellular cystine transport. Cystine is a precursor of glutathione, a key reducing metabolite that protects cells from ferroptosis.[41,42] Interestingly, in a gene set-enrichment analysis, we did not detect increased reactive oxygen species levels associated with the KUSS50 (data not shown). This suggests that ferroptosis activation in the benefit

subtype is driven not by heightened oxidative stress *per se*, but rather by increased susceptibility to lipid peroxidation, possibly due to elevated polyunsaturated fatty acid metabolism. Together, these findings support a model in which the benefit subtype is primed for ferroptosis and suggest that this vulnerability contributes to the enhanced efficacy of sorafenib. Further investigation into how the KUSS50 influences ferroptotic regulation and its therapeutic implications could offer valuable strategies for optimizing HCC treatment.

Despite showing a strong correlation of the KUSS50 with sorafenib benefit in HCC, our study had some limitations. First, the number of patients in the IMbrave150 sorafenib arm was small. This limitation primarily arises from the original genomic study's focus on identifying potential biomarkers of HCC response to the combination of atezolizumab and bevacizumab, resulting in the relatively limited availability of genomic data in the sorafenib arm.[20] Further validation of the KUSS50 in a larger cohort of patients given sorafenib is essential to ensure the robustness of the KUSS50 model. Second, the present study utilized data from a genomic substudy of the IMbrave150 trial, which included only a subset of patients with available genomic profiles. Therefore, the response rate differs from that of the original IMbrave150 trial, and the findings should be interpreted with caution. Validation using large-scale prospective genomic cohorts will be necessary to confirm these results. Third, although we explored associations of the KUSS50 with genomic subtypes of HCC, a deeper understanding of the functional consequences of these associations is necessary. Further investigations are required to elucidate the molecular pathways and processes through which the KUSS50 influences the benefit of sorafenib treatment in HCC. This knowledge could provide valuable information to identify potential therapeutic targets and strategies for improving the efficacy of sorafenib in patients with HCC. Fourth, while the KUSS50 was validated in the IMbrave150 and BIOSTORM cohorts, these cohorts may not fully capture the genetic and ethnic diversity of the global HCC population. Broader validation in more heterogeneous, geographically diverse populations will be necessary to confirm the generalizability and real-world applicability of the KUSS50. Finally, it is worth investigating the hypothesis that the KUSS50 also has predictive value for other multikinase inhibitors, such as regorafenib, which shares overlapping targets with sorafenib. Although promising, this hypothesis requires formal validation in patients treated with regorafenib or related agents to determine whether KUSS50 can serve as a broader predictive biomarker for this drug class.

In conclusion, our study highlights the clinical relevance of transcriptomic signatures in predicting sorafenib treatment outcomes in patients with HCC. The KUSS50 emerged as a promising tool for personalized medicine in HCC by predicting sorafenib benefit. The KUSS50 model is highly accurate and holds potential for optimizing treatment efficacy, minimizing side effects, and improving patient outcomes. Further research is warranted to explore the underlying biology of the KUSS50-defined subtypes of HCC, particularly the role of ferroptosis in HCC. To facilitate clinical translation, we refined the KUSS50 to a streamlined gene panel suitable for rapid and cost-effective platforms such as quantitative reverse-transcription PCR or NanoString. This enables practical application in routine clinical settings, in which gene expression profiling from pretreatment biopsies could be used to identify patients with high KUSS50 Bayesian probability scores (*e.g.* >0.7), who are more likely to respond favorably to sorafenib. Additionally, addressing the limitations of the KUSS50 model and investigating its applicability to other treatment options and diverse populations are crucial next steps. These findings present new avenues for improving the management of HCC through more precise molecular classification and better treatment guidance.

Affiliations

[1]*Department of Internal Medicine, Korea University, College of Medicine, Seoul, South Korea;* [2]*Department of Pathology, Korea University, College of Medicine, Seoul, South Korea;* [3]*Department of Internal Medicine, Hallym University Sacred Heart Hospital, Anyang, South Korea;* [4]*Department of Surgery, Korea University, College of Medicine, Seoul, South Korea;* [5]*Department of Biostatistics, Korea University, College of Medicine, Seoul, South Korea;* [6]*Department of Bioinformatics and Computational Biology, The University of Texas MD Anderson Cancer Center, Houston, Texas, USA;* [7]*Department of Systems Biology, The University of Texas MD Anderson Cancer Center, Houston, Texas, USA*

Abbreviations

HCC, hepatocellular carcinoma; KU, Korea University; KUSS50, Korea University Sorafenib Signature with 50 genes; NCIP, National Cancer Institute proliferation; ROC, receiver-operating characteristic; TCGA, The Cancer Genome Atlas.

Financial support

This work was supported by the NIH/NCI under award numbers R01CA237327, P50CA217674, and P30CA016672; the Duncan Family Institute for Cancer Prevention and Risk Assessment Seed Funding Research Program at MD Anderson (2016 cycle); institutional bridge funds from MD Anderson (2022 cycle); an Institutional Research Grant from MD Anderson (2021 cycle), to J.-S.L.; funds from The Research Supporting Program of The Korean Association for the Study of the Liver and The Korean Liver Foundation, to J.H.K.; and a grant from Industrial Strategic Technology Development Program (20024893, Development of non-thermal dynamic focusing focused ultrasound therapy device), funded by the Ministry of Trade, Industry and Energy, Korea, to S.Y.Y.

Conflict of interest

The authors declare no conflict of interest.

Please refer to the accompanying ICMJE disclosure forms for further details.

Authors' contributions

J.-S.L., J.H.K., and S.Y.Y. conceived and supervised the study. Y.-S.L., Y.K.J., Y.S.S., H.J.Y., B.S., and H.P. analyzed the data and prepared the figures. H.K., S.-H.K., E.C., Y.J.Y., S.H.K., J.E.Y., K.S.Y., S.H.L., Y.S.J., Y.T., T.H.K., Y.L., and H.L. processed and maintained data for analysis. S.Y.Y., J.H.K., and J.-S.L. wrote the manuscript and prepared the figures. All authors read and approved the final manuscript.

Data availability

The RNA sequencing data generated from the Korea University HCC cohort have been deposited in the NCBI Gene Expression Omnibus (GEO) under accession number GSE285625. Gene expression and corresponding clinical data from the IMbrave150 and GO30140 clinical trial cohorts were obtained from Genentech and are available through the European Genome-Phenome Archive (EGA) under accession number EGAD00001008128. Data from the BIOSTORM cohort, derived from the biomarker substudy of the phase III STORM trial, are publicly available in the GEO database under accession number GSE109211.

Acknowledgements

The authors thank Ashli Nguyen-Villarreal, Associate Scientific Editor, Don Norwood, Scientific Editor, and Joseph A Munch, Senior Scientific Editor, of the

Research Medical Library at The University of Texas MD Anderson Cancer Center for editing this manuscript.

Supplementary data

Supplementary data to this article can be found online at https://doi.org/10.1016/j.jhepr.2026.101742.

References

Author names in bold designate shared co-first authorship

[1] Sung H, Ferlay J, Siegel RL, et al. Global Cancer Statistics 2020: GLOBOCAN estimates of incidence and mortality worldwide for 36 cancers in 185 countries. CA Cancer J Clin 2021;71:209–249.
[2] Rumgay H, Arnold M, Ferlay J, et al. Global burden of primary liver cancer in 2020 and predictions to 2040. J Hepatol 2022;77:1598–1606.
[3] European Association for the Study of the Liver. EASL clinical practice guidelines: management of hepatocellular carcinoma. J Hepatol 2018;69:182–236.
[4] Cho Y, Kim BH, Park JW. Overview of Asian clinical practice guidelines for the management of hepatocellular carcinoma: an Asian perspective comparison. Clin Mol Hepatol 2023;29:252–262.
[5] Goh MJ, Sinn DH, Kim JM, et al. Clinical practice guideline and real-life practice in hepatocellular carcinoma: a Korean perspective. Clin Mol Hepatol 2023;29:197–205.
[6] Budai BK, Tóth A, Borsos P, et al. Three-dimensional CT texture analysis of anatomic liver segments can differentiate between low-grade and high-grade fibrosis. BMC Med Imaging 2020;20:108.
[7] Siegel RL, Giaquinto AN, Jemal A. Cancer statistics, 2024. CA Cancer J Clin 2024;74:12–49.
[8] Llovet JM, Ricci S, Mazzaferro V, et al. Sorafenib in advanced hepatocellular carcinoma. N Engl J Med 2008;359:378–390.
[9] Cheng AL, Kang YK, Chen Z, et al. Efficacy and safety of sorafenib in patients in the Asia-Pacific region with advanced hepatocellular carcinoma: a phase III randomised, double-blind, placebo-controlled trial. Lancet Oncol 2009;10:25–34.
[10] Wilhelm S, Carter C, Lynch M, et al. Discovery and development of sorafenib: a multikinase inhibitor for treating cancer. Nat Rev Drug Discov 2006;5:835–844.
[11] Ray EM, Sanoff HK. Optimal therapy for patients with hepatocellular carcinoma and resistance or intolerance to sorafenib: challenges and solutions. J Hepatocell Carcinoma 2017;4:131–138.
[12] Wörns MA, Galle PR. Sorafenib for the treatment of hepatocellular carcinoma. Hepat Oncol 2014;1:189–204.
[13] Sankar K, Gong J, Osipov A, et al. Recent advances in the management of hepatocellular carcinoma. Clin Mol Hepatol 2024;30:1–15.
[14] Chon YE, Kim DY, Kim MN, et al. Sorafenib vs. Lenvatinib in advanced hepatocellular carcinoma after atezolizumab/bevacizumab failure: a real-world study. Clin Mol Hepatol 2024;30:345–359.
[15] Miyahara K, Nouso K, Morimoto Y, et al. Pro-angiogenic cytokines for prediction of outcomes in patients with advanced hepatocellular carcinoma. Br J Cancer 2013;109:2072–2078.
[16] Horwitz E, Stein I, Andreozzi M, et al. Human and mouse VEGFA-amplified hepatocellular carcinomas are highly sensitive to sorafenib treatment. Cancer Discov 2014;4:730–743.
[17] Peer CJ, Sissung TM, Kim A, et al. Sorafenib is an inhibitor of UGT1A1 but is metabolized by UGT1A9: implications of genetic variants on pharmacokinetics and hyperbilirubinemia. Clin Cancer Res 2012;18:2099–2107.
[18] Finn RS, Qin S, Ikeda M, et al. Atezolizumab plus bevacizumab in unresectable hepatocellular carcinoma. N Engl J Med 2020;382:1894–1905.
[19] Lee MS, Ryoo BY, Hsu CH, et al. Atezolizumab with or without bevacizumab in unresectable hepatocellular carcinoma (GO30140): an open-label, multicentre, phase 1b study. Lancet Oncol 2020;21:808–820.
[20] Zhu AX, Abbas AR, de Galarreta MR, et al. Molecular correlates of clinical response and resistance to atezolizumab in combination with bevacizumab in advanced hepatocellular carcinoma. Nat Med 2022;28:1599–1611.
[21] Cheng AL, Qin S, Ikeda M, et al. Updated efficacy and safety data from IMbrave150: atezolizumab plus bevacizumab vs. sorafenib for unresectable hepatocellular carcinoma. J Hepatol 2022;76:862–873.
[22] Eisenhauer EA, Therasse P, Bogaerts J, et al. New response evaluation criteria in solid tumours: revised RECIST guideline (version 1.1). Eur J Cancer 2009;45:228–247.
[23] Bruix J, Takayama T, Mazzaferro V, et al. Adjuvant sorafenib for hepatocellular carcinoma after resection or ablation (STORM): a phase 3, randomised, double-blind, placebo-controlled trial. Lancet Oncol 2015;16:1344–1354.
[24] Pinyol R, Montal R, Bassaganyas L, et al. Molecular predictors of prevention of recurrence in HCC with sorafenib as adjuvant treatment and prognostic factors in the phase 3 STORM trial. Gut 2019;68:1065–1075.
[25] Lee JS, Chu IS, Heo J, et al. Classification and prediction of survival in hepatocellular carcinoma by gene expression profiling. Hepatology 2004;40:667–676.
[26] Lee JS, Heo J, Libbrecht L, et al. A novel prognostic subtype of human hepatocellular carcinoma derived from hepatic progenitor cells. Nat Med 2006;12:410–416.
[27] Kim SM, Leem SH, Chu IS, et al. Sixty-five gene-based risk score classifier predicts overall survival in hepatocellular carcinoma. Hepatology 2012;55:1443–1452.
[28] **Lee SH**, **Yim SY**, Jeong YS, et al. Consensus subtypes of hepatocellular carcinoma associated with clinical outcomes and genomic phenotypes. Hepatology 2022;76:1634–1648.
[29] Lee SH, Jeong YS, Lee S, et al. Two distinct stem cell-like subtypes of hepatocellular carcinoma with clinical significance and their therapeutic potentials. Cancer Commun (Lond) 2022;42:179–183.
[30] Jung J, Park S, Jang Y, et al. Clinical significance of glycolytic metabolic activity in hepatocellular carcinoma. Cancers (Basel) 2022;15:186.
[31] TCGA_consortium. Comprehensive and integrative genomic characterization of hepatocellular carcinoma. Cell 2017;169:1327–1341. https://doi.org/10.1016/j.cell.2017.05.046. e23 LID - S0092-8674(17)30639-6 [pii] LID -.
[32] Simon R, Lam A, Li MC, et al. Analysis of gene expression data using BRB-ArrayTools. Cancer Inform 2007;3:11–17.
[33] Eisen MB, Spellman PT, Brown PO, et al. Cluster analysis and display of genome-wide expression patterns. Proc Natl Acad Sci U S A 1998;95:14863–14868.
[34] Yoshihara K, Shahmoradgoli M, Martínez E, et al. Inferring tumour purity and stromal and immune cell admixture from expression data. Nat Commun 2013;4:2612.
[35] Carter SL, Cibulskis K, Helman E, et al. Absolute quantification of somatic DNA alterations in human cancer. Nat Biotechnol 2012;30:413–421.
[36] Liu Y, Ye X, Zhang JB, et al. PROX1 promotes hepatocellular carcinoma proliferation and sorafenib resistance by enhancing β-catenin expression and nuclear translocation. Oncogene 2015;34:5524–5535.
[37] Leung HW, Leung CON, Lau EY, et al. EPHB2 activates β-catenin to enhance cancer stem cell properties and drive sorafenib resistance in hepatocellular carcinoma. Cancer Res 2021;81:3229–3240.
[38] Deng L, Sun J, Chen X, et al. Nek2 augments sorafenib resistance by regulating the ubiquitination and localization of β-catenin in hepatocellular carcinoma. J Exp Clin Cancer Res 2019;38:316.
[39] Hoshida Y, Nijman SM, Kobayashi M, et al. Integrative transcriptome analysis reveals common molecular subclasses of human hepatocellular carcinoma. Cancer Res 2009;69:7385–7392.
[40] Desert R, Rohart F, Canal F, et al. Human hepatocellular carcinomas with a periportal phenotype have the lowest potential for early recurrence after curative resection. Hepatology 2017;66:1502–1518.
[41] Dixon SJ, Lemberg KM, Lamprecht MR, et al. Ferroptosis: an iron-dependent form of nonapoptotic cell death. Cell 2012;149:1060–1072.
[42] Stockwell BR, Friedmann Angeli JP, Bayir H, et al. Ferroptosis: a regulated cell death nexus linking metabolism, redox biology, and disease. Cell 2017;171:273–285.
[43] Doll S, Proneth B, Tyurina YY, et al. ACSL4 dictates ferroptosis sensitivity by shaping cellular lipid composition. Nat Chem Biol 2017;13:91–98.
[44] Kagan VE, Mao G, Qu F, et al. Oxidized arachidonic and adrenic PEs navigate cells to ferroptosis. Nat Chem Biol 2017;13:81–90.
[45] Lee JY, Nam M, Son HY, et al. Polyunsaturated fatty acid biosynthesis pathway determines ferroptosis sensitivity in gastric cancer. Proc Natl Acad Sci U S A 2020;117:32433–32442.

[46] Belletti B, Baldassarre G. Stathmin: a protein with many tasks. New biomarker and potential target in cancer. Expert Opin Ther Targets 2011;15:1249–1266.
[47] Correia AL, Bissell MJ. The tumor microenvironment is a dominant force in multidrug resistance. Drug Resist Updat 2012;15:39–49.
[48] Hou J, Jiang C, Wen X, et al. ACSL4 as a potential target and biomarker for anticancer: from molecular mechanisms to clinical therapeutics. Front Pharmacol 2022;13:949863.
[49] Louandre C, Ezzoukhry Z, Godin C, et al. Iron-dependent cell death of hepatocellular carcinoma cells exposed to sorafenib. Int J Cancer 2013;133:1732–1742.
[50] Lachaier E, Louandre C, Godin C, et al. Sorafenib induces ferroptosis in human cancer cell lines originating from different solid tumors. Anticancer Res 2014;34:6417–6422.

Keywords: Markers; Transcriptome; IMbrave150; Personalized treatment; Ferroptosis.
Received 21 March 2025; received in revised form 7 January 2026; accepted 14 January 2026; Available online 27 January 2026

Journal of Hepatology, Volume *8*

Supplemental information

Identifying sorafenib benefit among patients with hepatocellular carcinoma: A transcriptomic and genomic approach

Sun Young Yim, Hayeon Kim, Tae Hyung Kim, Sang-Hee Kang, Youngwoo Lee, Eunho Choi, Yang Jae Yoo, Seong Hee Kang, Young-Sun Lee, Young Kul Jung, Yeon Seok Seo, Hyung Joon Yim, Jong Eun Yeon, Kyung Suk Yang, Yitao Tang, Bowha Sohn, Yun Seong Jeong, Hyewon Park, Han Liang, Ju-Seog Lee, and Ji Hoon Kim

Identifying sorafenib benefit among patients with hepatocellular carcinoma: A transcriptomic and genomic approach

Sun Young Yim, Hayeon Kim, Tae Hyung Kim, Sang-Hee Kang, Youngwoo Lee, Eunho Choi, Yang Jae Yoo, Seong Hee Kang, Young-Sun Lee, Young Kul Jung, Yeon Seok Seo, Hyung Joon Yim, Jong Eun Yeon, Kyung Suk Yang, Yitao Tang, Bowha Sohn, Yun Seong Jeong, Hyewon Park, Han Liang, Ju-Seog Lee, Ji Hoon Kim

Table of contents

Fig. S1……………………………………………………………………………………2
Fig. S2……………………………………………………………………………………3
Fig. S3……………………………………………………………………………………4
Fig. S4……………………………………………………………………………………5
Fig. S5……………………………………………………………………………………6
Fig. S6……………………………………………………………………………………7
Fig. S7……………………………………………………………………………………8
Fig. S8……………………………………………………………………………………9
Fig. S9……………………………………………………………………………………10
Table S1…………………………………………………………………………………11
Table S2…………………………………………………………………………………13
Table S3…………………………………………………………………………………15
Table S4…………………………………………………………………………………16
Table S5…………………………………………………………………………………17
Table S6…………………………………………………………………………………18

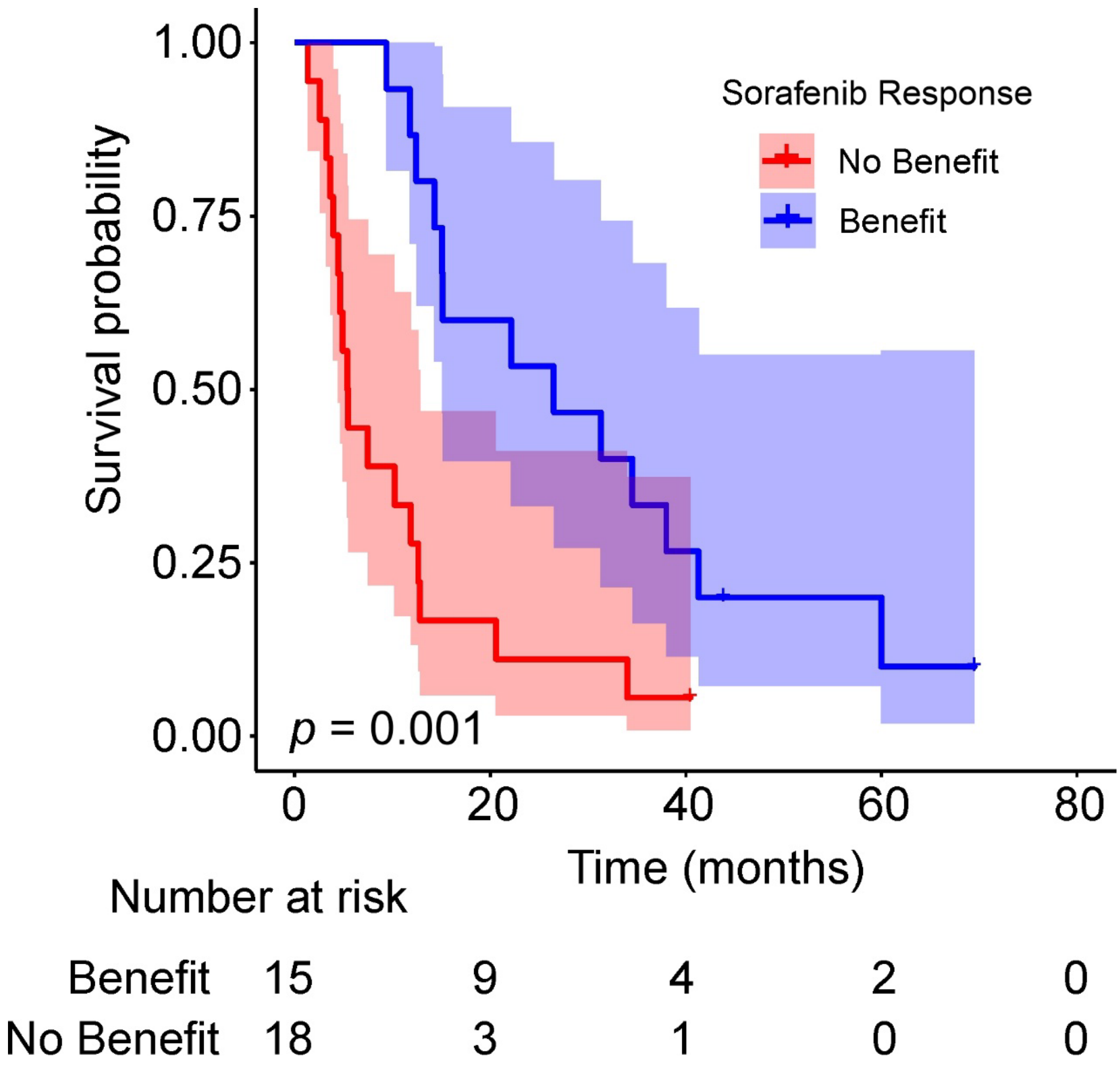

Fig. S1. Overall survival differs significantly between the sorafenib benefit and non-benefit groups in the KU cohort.

Kaplan–Meier curves comparing overall survival (OS) between patients in the KU cohort classified as having a sorafenib benefit (n = 15) or no sorafenib benefit (n = 18) based on clinical response to sorafenib treatment. The benefit group included patients who did not experience disease progression while receiving sorafenib, whereas the non-benefit group comprised patients whose disease progressed despite treatment. A significant difference in OS was observed between the two groups (log-rank p = 0.001), with the benefit group showing markedly improved survival. These findings highlight the clinical importance of distinguishing patients who derive benefit from sorafenib and provide a basis for developing predictive gene expression signatures.

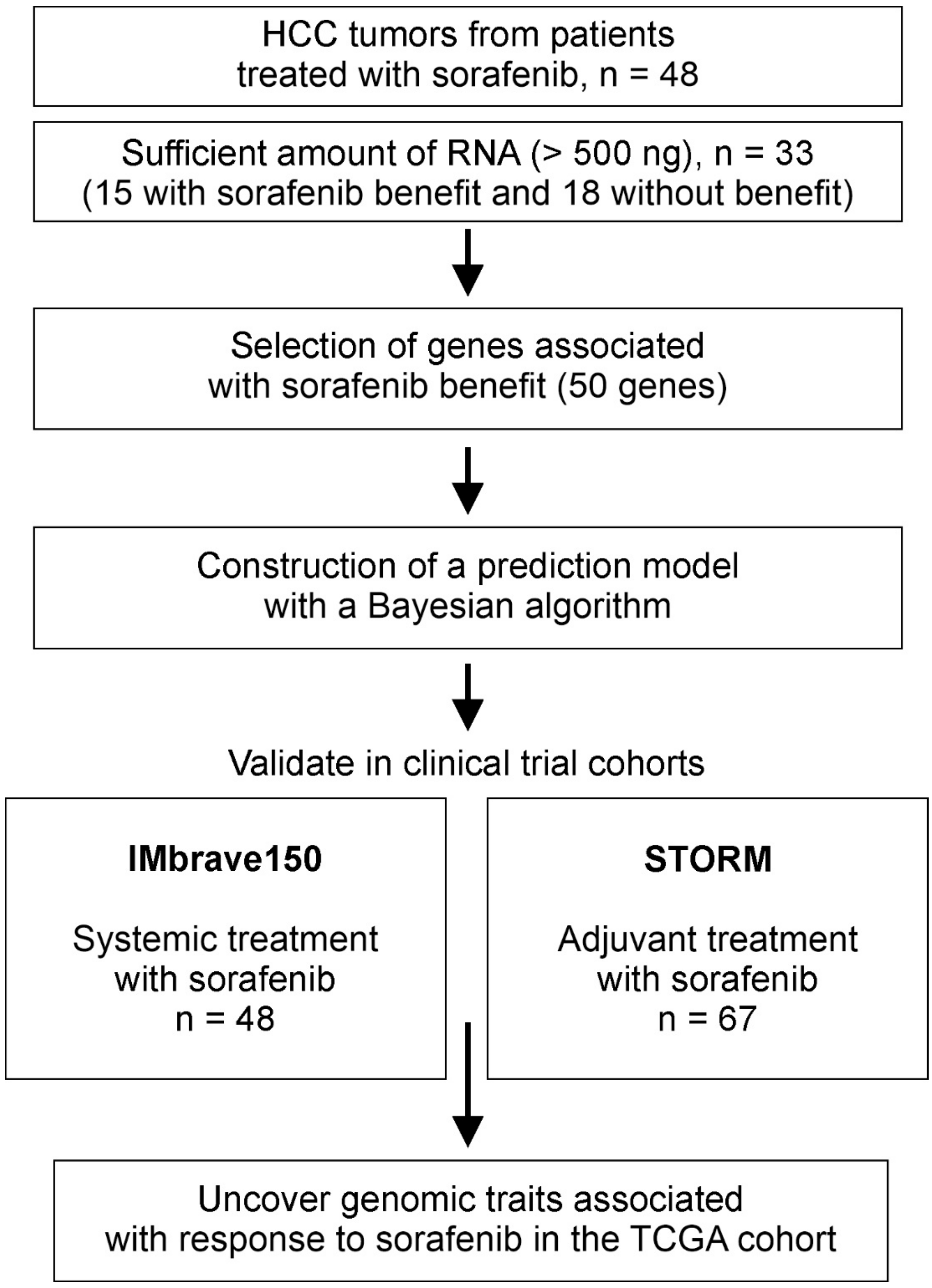

Fig. S2. Schematic of the data analysis for the identification and confirmation of predictive biomarkers in sorafenib-treated HCC patients.

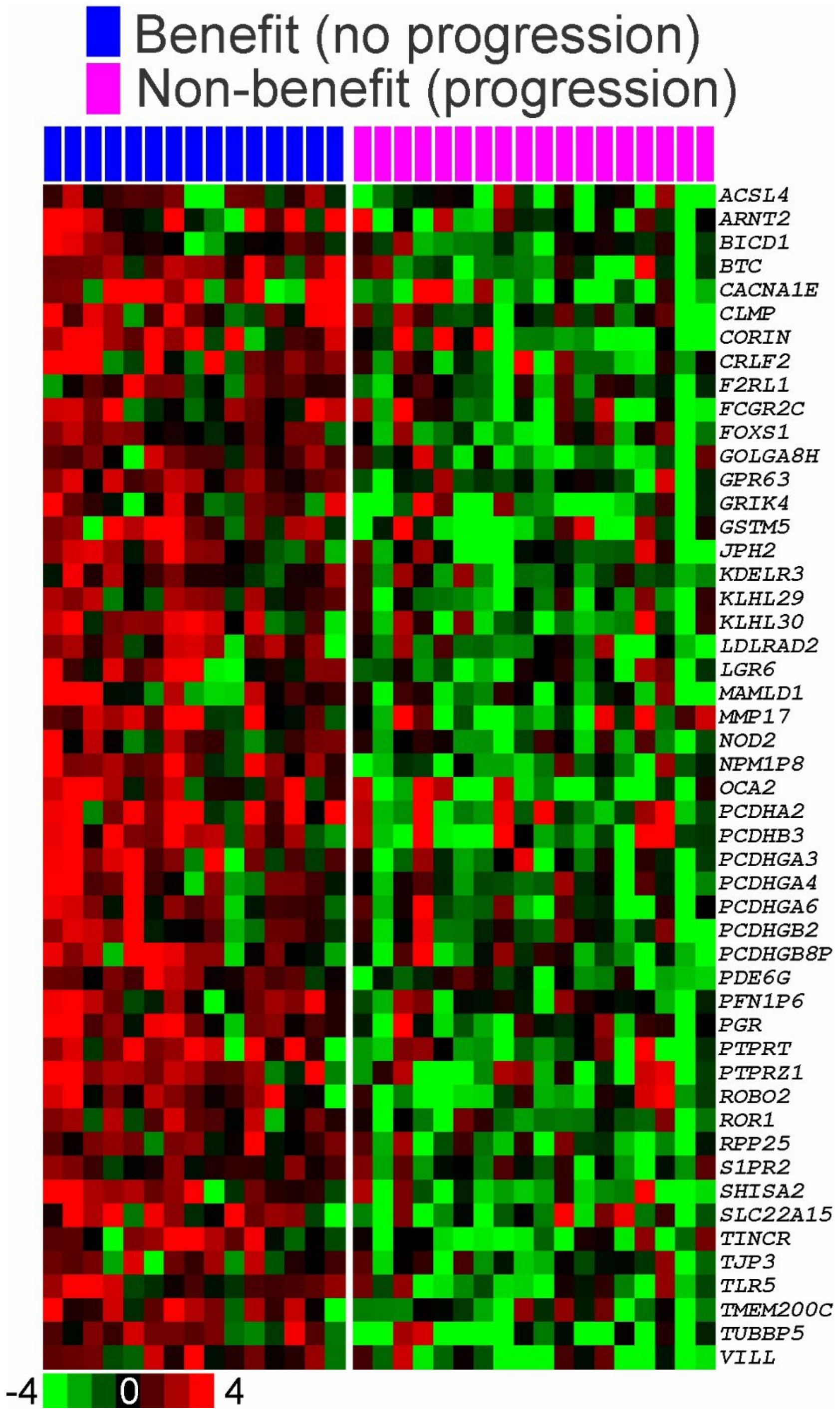

Fig. S3. Expression patterns of the genes included in the KUSS50.

The data are presented in matrix format; each row represents an individual gene, and each column represents a different tissue sample. Each cell in the matrix represents the expression level of a gene in an individual tissue sample. The red and green colors in the cells reflect relatively high and low gene expression levels, respectively, as indicated by the scale bar (log2-transformed scale).

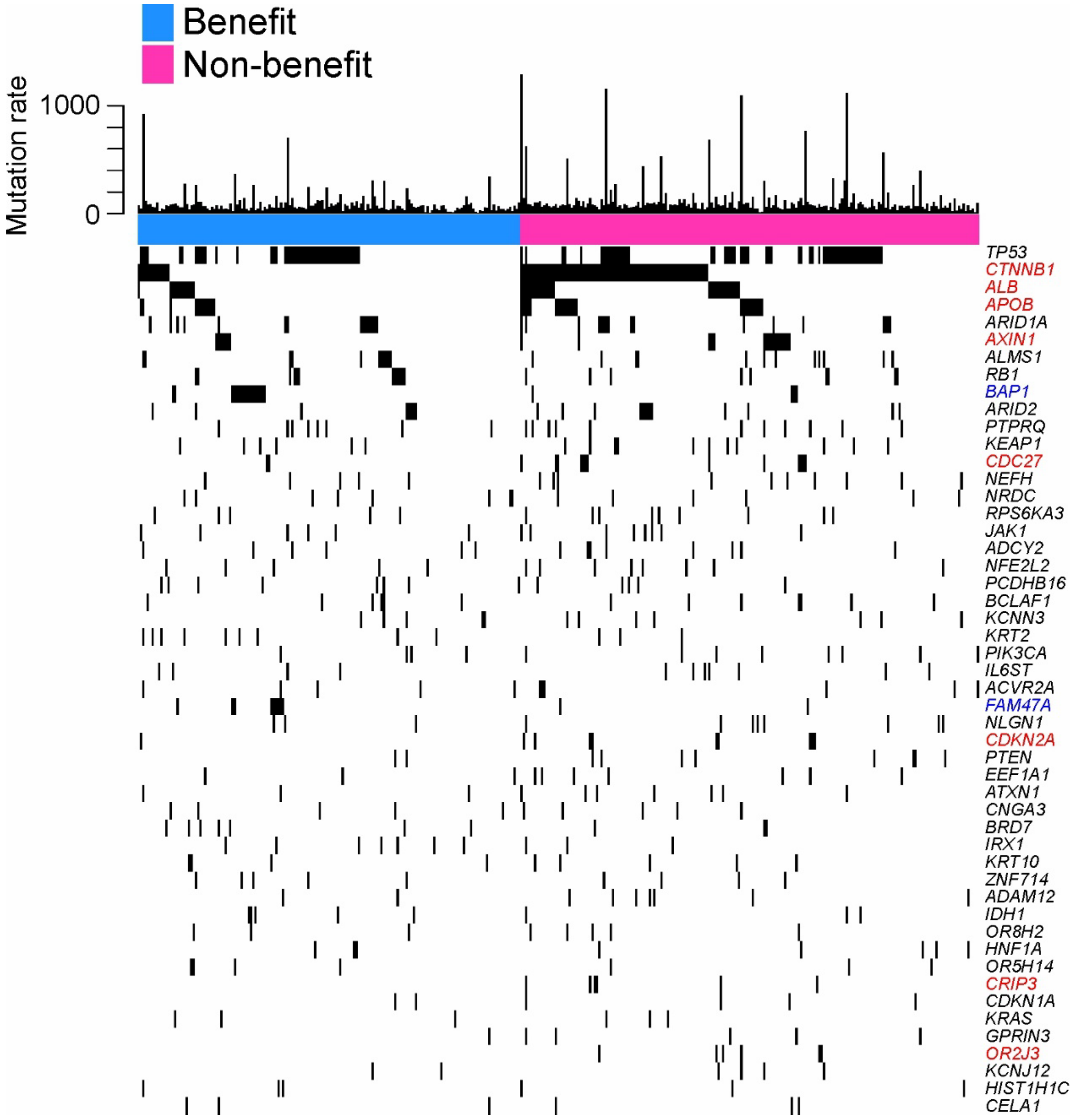

Fig. S4. Profile of the somatic mutations in the KUSS50-defined subtypes in the TCGA cohort. The top panel displays the individual tumor mutation rates for each patient in the TCGA cohort. Each horizontal bar represents a single tumor sample, with the height of the bar indicating the overall mutation rate (or tumor mutation burden) for that tumor. The bottom panel shows the mutation landscape, with genes exhibiting significant levels of mutation in the TCGA cohort highlighted. The genes displayed in this panel met two criteria: 1) a false discovery rate (FDR) of less than 0.1, as determined via MutSig suite analysis, and 2) a mutation rate greater than 7% across the cohort. The gene symbols highlighted in red indicate genes that are significantly associated with the non-benefit subtype, as determined by a chi-square test ($p < 0.05$). Conversely, the gene symbols highlighted in blue represent genes significantly associated with the benefit subtype.

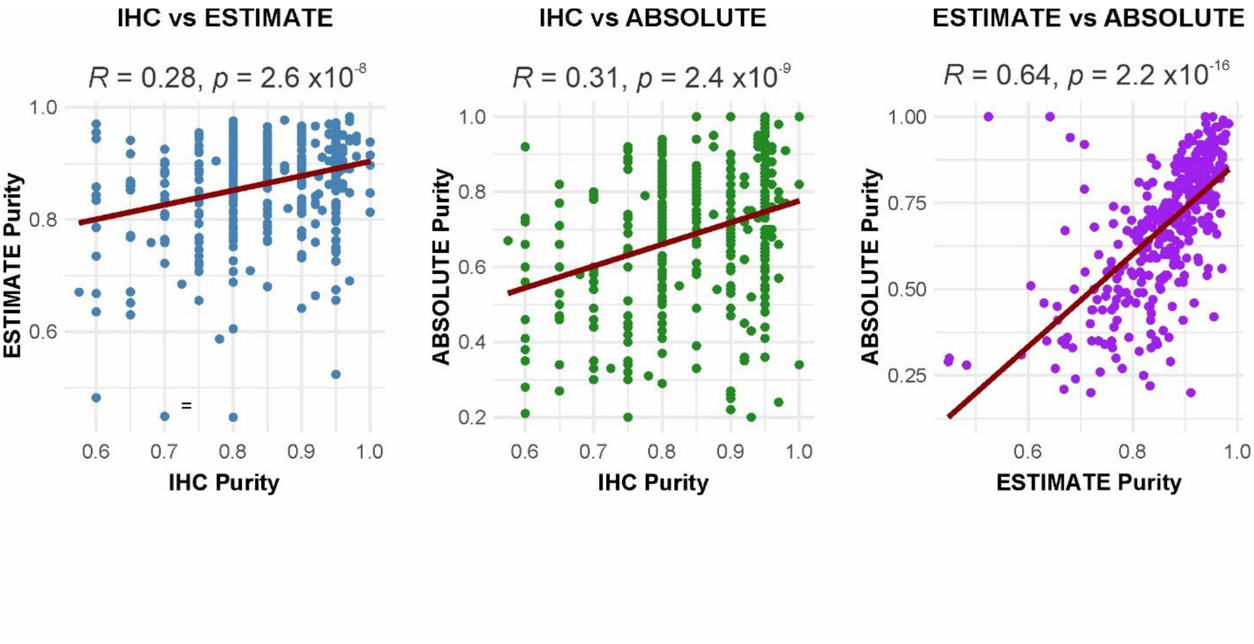

Fig. S5. Correlation among the three independent methods used to estimate the purity of HCC tumors from the TCGA cohort.

The strength and significance of the correlations were assessed using the Pearson correlation test, with corresponding *R* and *p*-values indicated on each plot.

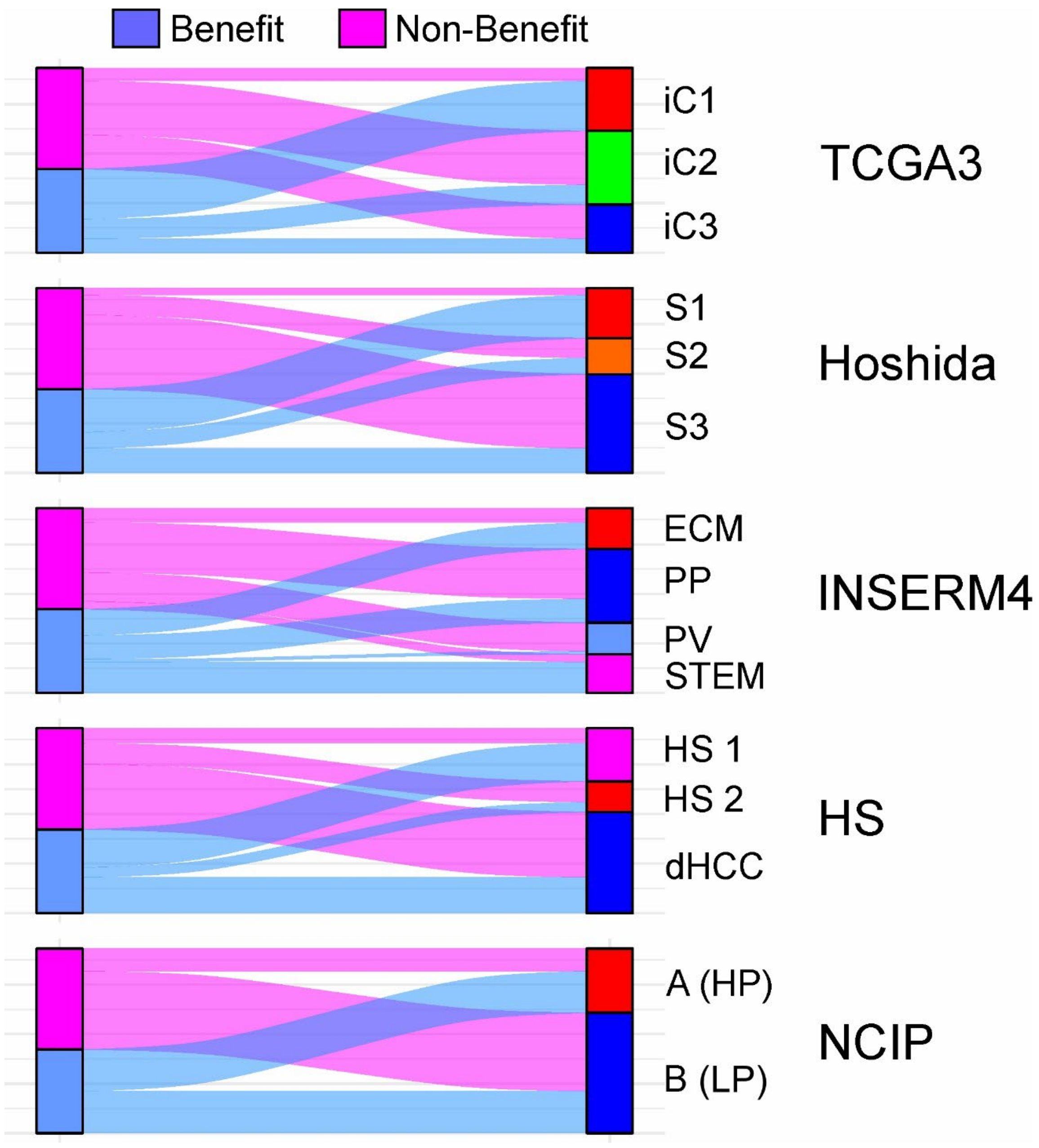

Fig. S6. Sankey plots of the KUSS50-defined subtypes compared with genomic subtypes previously identified in HCC patients.

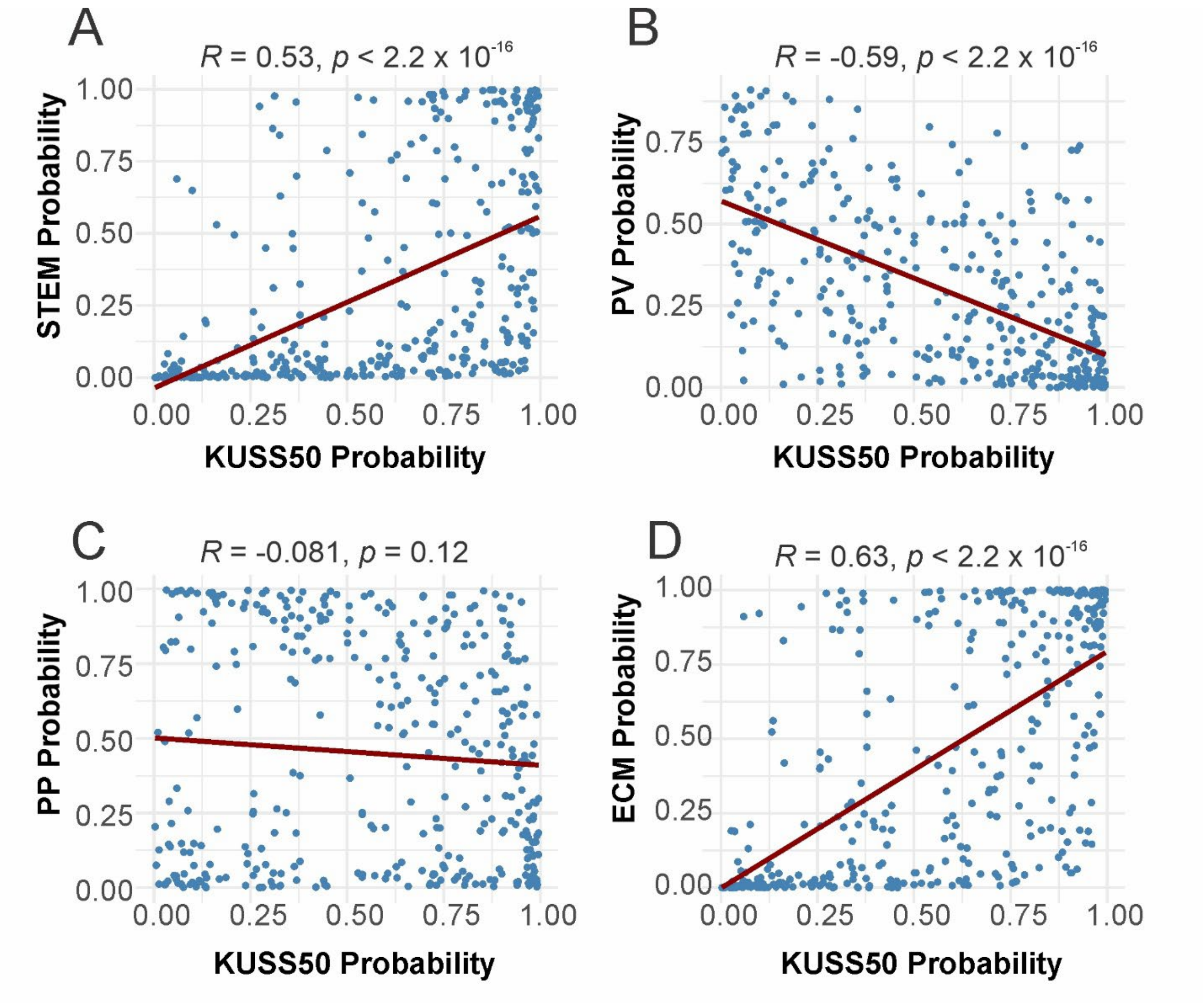

Fig. S7. Correlation of KUSS50-based subtype probability with the probabilities of four INSERM molecular subtypes in HCC.

(A) STEM (stem cell), (B) PV (perivenous), (C) PP (periportal), and (D) ECM (extracellular matrix) subtype probabilities were plotted against KUSS50 probability scores. Each dot represents an individual HCC tumor. The red line indicates the linear regression (lm) fit, and Pearson correlation coefficients (*R*) with associated *p*-values are shown. Significant positive correlations were observed between the KUSS50-defined subtype and the STEM subtype (R = 0.53) and ECM subtype (R = 0.63), while a significant negative correlation was seen with the PV subtype (R = -0.59). No significant correlation was found with the PP subtype. Statistical significance was estimated by the Pearson correlation test.

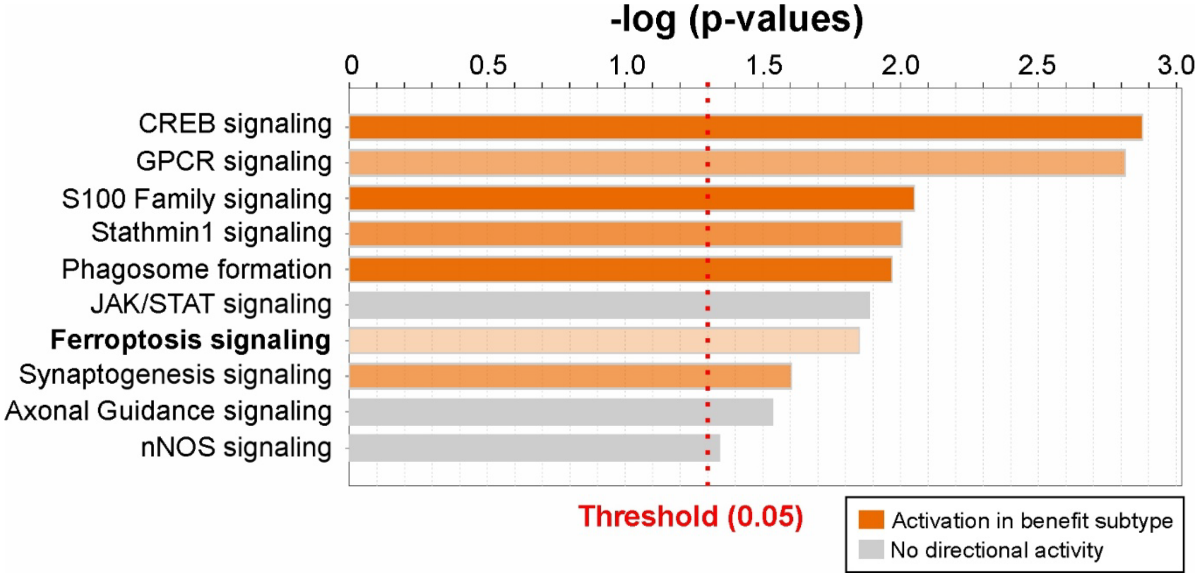

Fig. S8. Ingenuity Pathway Analysis (IPA) identifies the canonical pathways of genes in the KUSS50.

The x-axis represents the –log(p) value of pathway enrichment, and the y-axis lists the 10 most significantly enriched canonical pathways among genes upregulated in the KUSS50-defined benefit subtype. Each bar corresponds to a canonical pathway, with its length indicating the level of statistical significance of enrichment. Pathway enrichment significance was determined using the right-tailed Fisher exact test; longer bars indicate stronger enrichment, i.e., higher –log(p) values. The color of each bar denotes the activation *z*-score, reflecting predicted activation (orange), inhibition (blue), or undetermined directionality (gray) based on expression patterns. Pathways with $p < 0.05$ were considered significantly enriched.

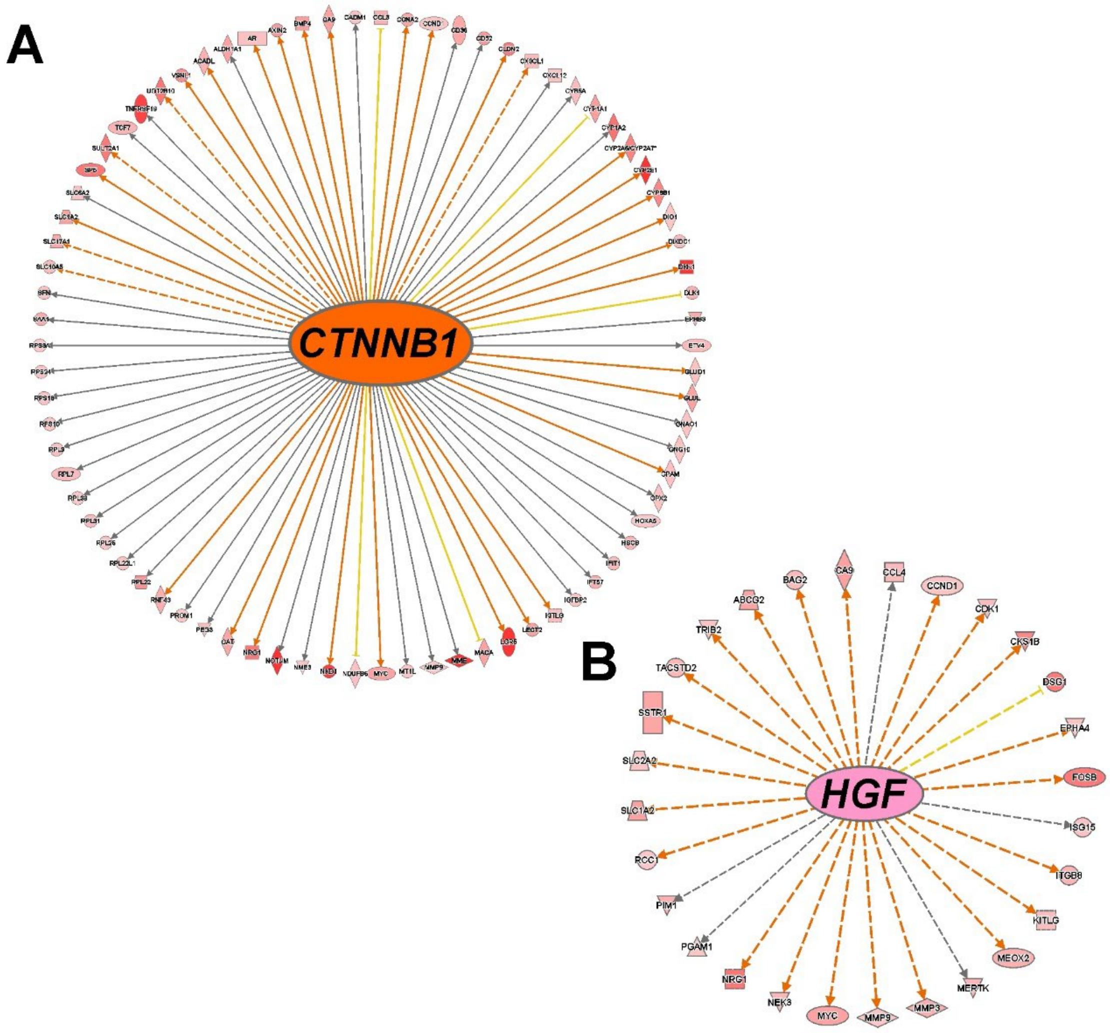

Fig. S9. Gene networks of upstream regulators activated in the non-benefit subtype of HCC.
Upstream regulator analysis was performed using Ingenuity Pathway Analysis (IPA) of the genes upregulated in the non-benefit subtype of HCC. Shown here are the gene networks centered on key upstream regulators predicted to be significantly activated based on the *z*-scores and expression patterns of downstream targets. (A) The CTNNB1 (β-catenin) network showing a broad range of transcriptional targets, reflecting its central role in Wnt signaling and previously known association with sorafenib resistance. (B) The HGF (hepatocyte growth factor) network illustrating the activation of mitogenic and pro-survival signaling pathways. Node colors represent expression changes (e.g., red for upregulated targets), and edge colors indicate the direction and type of regulation inferred by IPA. Solid and dashed lines represent direct and indirect relationships, respectively.

Table S1. Clinical characteristics of HCC patients treated with sorafenib.

Clinical characteristic	Total	Benefit	Non-benefit	*p*-value
**No. of patients**	33	15	18	
**Age, years, median (range)**	58 (32-83)	58 (41-71)	57 (32-83)	0.915
**Gender, male, *N* (%)**	27 (81.8)	12 (80)	15 (83.3)	0.805
**Cirrhosis, *N* (%)**	19 (57.6)	8 (53.3)	11 (61.1)	0.653
**AST, IU/L, median (range)**	55 (19-317)	41 (19-157)	38 (19-317)	0.957
**ALT, IU/L, median (range)**	29 (7-211)	29 (14-90)	29 (7-211)	0.492
**Total bilirubin, mg/dL, median (range)**	0.6 (0.33-5.07)	0.59 (0.36-2.02)	0.7 (0.33-5.07)	0.575
**Albumin, median (range)**	2.9 (2.9-4.6)	4.2 (3.1-4.4)	4.0 (2.9-4.6)	0.284
**INR, median (range)**	1.03 (0.89-1.23)	1.02 (0.89-1.19)	1.05 (0.92-1.23)	0.302
**CTP grade**				0.538
**5**	25	14 (77.8)	11 (73.3)	
**6**	7	3 (16.7)	4 (26.7)	
**8**	1	1 (5.6)	0	
**ALBI score, median (range)**	-2.74 (-3.23- -1.44)	-2.80 (-3.21- -1.82)	-2.67 (-3.23- -1.44)	0.29
**Log_{10} AFP, median (range)**	2.17 (0.04-6.28)	1.76 (0.34-6.28)	2.46 (0.04-5.68)	0.708
**Log_{10}PIVKA, median (range)**	2.33 (0.95-5)	1.58 (0.95-5.00)	2.46 (1.08-5.00)	0.156
**Etiology, *N* (%)**				
HBV	24 (72.7)	12 (80)	12 (66.7)	0.539
HCV	1 (3)	0	1 (5.6)	
Nonviral	8 (24.2)	3 (20)	5 (27.8)	
**Extrahepatic metastasis, *N* (%)**	29 (87.9)	12 (80)	17 (94.4)	0.206
**Vessel invasion, *N* (%)**				
Major portal vein	8 (24.2)	3 (20)	5 (27.8)	0.604
Major hepatic vein	3 (9.1)	2 (13.3)	1 (5.6)	0.439
**Performance status, *N* (%)**				0.730

0	23 (69.7)	10 (66.7)	13 (72.2)	
1	10 (30.3)	5 (33.3)	5 (27.8)	
**BCLC stage, *N* (%)**				0.894
B	2 (6.1)	1 (6.7)	1 (5.6)	
C	31 (93.9)	14 (93.3)	17 (94.4)	
**Dermatologic adverse event, *N* (%)**	17 (51.5)	11(73.3)	6 (33.3)	0.022
**2nd-line CTx, *N* (%)**				
None	14 (42.4)	7 (46.7)	7 (38.9)	0.566
APX	8 (24.2)	4 (26.7)	4 (22.2)	
EPUL	4 (12.1)	1 (6.7)	3 (16.7)	
Nivolumab	2 (6.1)	0	2 (11.1)	
Regorafenib	5 (15.2)	3 (20)	2 (11.1)	

AST, aspartate aminotransferase; ALT, alanine aminotransferase; INR, international normalized ratio; CTP, Child-Pugh Score; ALBI, albumin-bilirubin; AFP, alpha-fetoprotein; PIVKA, proteins induced by vitamin K absence or antagonism-II; HBV, hepatitis B virus; HCV, hepatitis C virus; BCLC, Barcelona Clinic Liver Cancer; CTx, chemotherapy; APX, adriamycin + capecitabine; EPUL, epirubicin, cisplatin, UFT, and leucovorin

Table S2. Genes in the KUSS50.

EnsEMBL gene ID	Symbol	Ratio (benefit/non-benefit)
ENSG00000068366	*ACSL4*	1.69
ENSG00000172379	*ARNT2*	2.09
ENSG00000151746	*BICD1*	1.04
ENSG00000174808	*BTC*	2.10
ENSG00000198216	*CACNA1E*	2.58
ENSG00000166250	*CLMP*	2.01
ENSG00000145244	*CORIN*	2.37
ENSG00000205755	*CRLF2*	1.72
ENSG00000164251	*F2RL1*	1.20
ENSG00000244682	*FCGR2C*	1.55
ENSG00000179772	*FOXS1*	1.51
ENSG00000261794	*GOLGA8H*	1.72
ENSG00000112218	*GPR63*	1.74
ENSG00000149403	*GRIK4*	2.28
ENSG00000134201	*GSTM5*	2.27
ENSG00000149596	*JPH2*	1.61
ENSG00000100196	*KDELR3*	1.15
ENSG00000119771	*KLHL29*	1.74
ENSG00000168427	*KLHL30*	1.78
ENSG00000187942	*LDLRAD2*	1.67
ENSG00000133067	*LGR6*	1.65
ENSG00000013619	*MAMLD1*	1.45
ENSG00000198598	*MMP17*	1.71
ENSG00000167207	*NOD2*	1.21
ENSG00000236285	*NPM1P8*	2.54
ENSG00000104044	*OCA2*	2.96
ENSG00000204969	*PCDHA2*	1.93
ENSG00000113205	*PCDHB3*	2.00
ENSG00000254245	*PCDHGA3*	1.98
ENSG00000262576	*PCDHGA4*	1.46
ENSG00000253731	*PCDHGA6*	1.42
ENSG00000253910	*PCDHGB2*	1.17
ENSG00000248449	*PCDHGB8P*	1.55
ENSG00000185527	*PDE6G*	1.95
ENSG00000227212	*PFN1P6*	1.45
ENSG00000082175	*PGR*	2.04
ENSG00000196090	*PTPRT*	2.21
ENSG00000106278	*PTPRZ1*	2.37
ENSG00000185008	*ROBO2*	1.76
ENSG00000185483	*ROR1*	1.56

ENSG00000178718	*RPP25*	1.70
ENSG00000267534	*S1PR2*	1.04
ENSG00000180730	*SHISA2*	2.35
ENSG00000163393	*SLC22A15*	1.83
ENSG00000223573	*TINCR*	2.01
ENSG00000105289	*TJP3*	1.13
ENSG00000187554	*TLR5*	2.11
ENSG00000206432	*TMEM200C*	1.65
ENSG00000159247	*TUBBP5*	2.85
ENSG00000136059	*VILL*	1.85

Table S3. Factors associated with benefit from sorafenib treatment.

Characteristic	Univariate		Multivariate	
	HR (95% CI)	*p*-value	HR (95% CI)	*p*-value
Age, years	1.011 (0.971-1.052)	0.606		
Male gender	0.507 (0.199-1.291)	0.154		
Liver cirrhosis	0.917 (0.446-1.882)	0.813		
CTP grade	1 (0.422-2.366)	1		
ALBI score	2.412 (0.957-6.078)	0.062		
Log_{10} AFP	1.206 (0.964-1.509)	0.101	1.348 (1.048-1.733)	0.02
Log_{10}PIVKA	1.302 (0.95-1.783)	0.1		
Etiology				
Non-HBV	Reference			
HBV	1.408 (0.639-3.102)	0.396		
Extrahepatic metastasis				
No	Reference			
Yes	2.709 (0.628-11.685)	0.181		
Major portal vein	0.874 (0.371-2.058)	0.757		
Major hepatic vein	1.025 (0.316-3.517)	0.935		
ECOG performance status				
0	Reference		Reference	
1	1.935 (0.884-4.238)	0.099	2.862 (1.193-6.863)	0.018
BCLC stage				
B	Reference			
C	1.416 (0.316-6.351)	0.649		
Skin adverse event	0.422 (0.199-0.894)	0.024		
KUSS50				
Benefit	Reference		Reference	
Non-benefit	0.288 (0.132-0.633)	0.002	0.181 (0.073-0.451)	<0.001

CTP, Child-Pugh Score; ALBI, albumin-bilirubin; AFP, alpha-fetoprotein; PIVKA, proteins induced by vitamin K absence or antagonism-II; HBV, hepatitis B virus; BCLC, Barcelona Clinic Liver Cancer

Table S4. Contingency table depicting the association of the KUSS50 with the benefit of atezolizumab treatment in the GO30140 cohort.

**Prediction**	**Partial response or better**	**Non-responder**	**Total**
Benefit	5	12	17
Non-benefit	4	22	26
Total	9	34	43

p = 0.48 by χ^2 test

Table S5. Two-way contingency table depicting the association of the KUSS50 with the benefit of atezolizumab and bevacizumab treatment in the IMbrave150 + GO30140 cohort.

Prediction	Partial response or better	Non-responder	Total
Benefit	28	56	84
Non-benefit	53	110	163
Total	81	166	247

p = 0.91 by χ^2 test

Table S6. Activated upstream regulators in the non-benefit subtype predicted by gene network analysis.

Upstream regulator	Molecular feature	Predicted state	Activation *z*-score	*p*-value	Genes
**MYC**	Transcription regulator	Activated	5.789	0.000831	64
**CTNNB1**	Transcription regulator	Activated	4.56	1.93E-13	59
**HNF1A**	Transcription regulator	Activated	4.196	6.22E-14	52
**HGF**	Growth factor	Activated	4.09	0.00144	28
**N6AMT1**	Enzyme	Activated	4.082	4.03E-06	17
**STAT3**	Transcription regulator	Activated	4.068	0.0139	37
**SP1**	Transcription regulator	Activated	4.053	0.000662	34
**RELA**	Transcription regulator	Activated	4.032	0.00701	26
**CD40**	Transmembrane receptor	Activated	3.908	0.015	26
**MYCN**	Transcription regulator	Activated	3.869	0.000159	24
**NFKB**	Transcription regulator	Activated	3.836	0.0127	30
**MLXIPL**	Transcription regulator	Activated	3.712	0.000202	14
**ERBB2**	Kinase	Activated	3.618	2.27E-07	64
**AHR**	Nuclear receptor	Activated	3.604	6.39E-07	41
**TCF7L2**	Transcription regulator	Activated	3.57	0.000162	28
**NFE2L2**	Transcription regulator	Activated	3.569	0.000053	43
**CD44**	Other	Activated	3.541	0.0045	16
**IL27**	Cytokine	Activated	3.537	0.0115	14
**ERK1/2**	Kinase	Activated	3.414	0.000393	22
**EPO**	Cytokine	Activated	3.372	0.0014	20

This table summarizes the results of the Ingenuity Pathway Analysis (IPA) of genes upregulated in the non-benefit subtype, highlighting predicted upstream regulators that may drive the observed transcriptional profile. Only the top 20 upstream regulators are shown, ranked by activation *z*-score.

Upstream regulators: The top 20 genes or molecules predicted to regulate downstream targets in the dataset.
Molecular features: The functional class of the regulator (transcription factor, kinase, cytokine, etc.).
Predicted state: Indicates whether the upstream regulator is predicted to be activated based on the direction of expression changes in its known targets.
Activation *z*-score: A statistical measure of regulator activity; scores >2 suggest activation with confidence, with higher values indicating stronger predicted activation.
***p*-value:** Statistical significance of the overlap between known targets of the regulator and the differentially expressed genes estimated by the Fisher exact test; lower values indicate stronger enrichment.
Genes: Number of genes in the dataset known to be regulated by the upstream regulator.
